# Sterol divergence across eukaryotic kingdoms determines membrane susceptibility to saponins, a class of plant defense compounds

**DOI:** 10.1073/pnas.2523859123

**Published:** 2026-05-08

**Authors:** Malbor Dervishi, Jan Günther, Jinhui Li, Huriye Deniz Uzun, Hans Christian Bruun Hansen, Thomas Günther Pomorski, Anja Thoe Fuglsang, Viviana Monje, Søren Bak

**Affiliations:** ^a^ Department of Plant and Environmental Sciences, University of Copenhagen, Frederiksberg 1871, Denmark; ^b^ Department of Chemical and Biological Engineering, University of Buffalo, Amherst, NY 14260; ^c^ Department of Molecular Biochemistry, Faculty of Chemistry and Biochemistry, Ruhr University Bochum, Bochum 44780, Germany

**Keywords:** saponins, sterol, plant defense, metabolites, co-evolution

## Abstract

Saponins are a class of plant-derived amphiphile defense compounds that disrupt cellular membranes, yet the basis for their selective toxicity remains unclear. Because membrane sterols diverged across the three major eukaryotic kingdoms from a shared biosynthetic precursor, we tested whether sterol identity governs membrane susceptibility to saponins. Using yeast, sterol supplementation, synthetic liposomes, and molecular dynamics simulations, we compared membranes containing zoosterols, mycosterols, or phytosterols. When a panel of structurally different saponins was tested, reduced ergosterol levels in yeast were consistently associated with decreased lytic activity. A similar reduction in susceptibility was observed when ergosterol was replaced by phytosterols in yeast. These trends were recapitulated in a simplified membrane system, where the sterol identity in large unilamellar vesicles strongly influenced saponin-induced lysis. The triterpenoid saponin α-hederin efficiently lysed membranes enriched in cholesterol or ergosterol, whereas membranes containing plant phytosterols were markedly resistant. In contrast, the steroidal saponin digitonin exhibited lower lytic activity and limited sterol selectivity. Molecular dynamic simulations revealed sterol-dependent clustering and membrane responses that paralleled experimental susceptibility. Together, these findings support a two-parameter model where structural characteristics of saponins together with sterol identity in the membrane are a primary determinant of saponin-induced membrane disruption. The differential compatibility between saponins and sterol classes provides a mechanistic framework for understanding cross-kingdom selectivity and sheds light on how plants avoid self-toxicity while deploying saponins for defense.

## Significance

The basis for the selective activity of saponins across organisms, and for plant self-resistance during their biosynthesis and storage, is not fully understood. Here, we show that membrane sterol identity governs susceptibility to saponins and propose a two-parameter framework in which both saponin structural characteristics and membrane sterol composition determine membrane disruption. This model provides a mechanistic explanation for cross-kingdom selectivity and offers insight into how plants deploy saponins while avoiding self-toxicity.

**Fig. 1.**
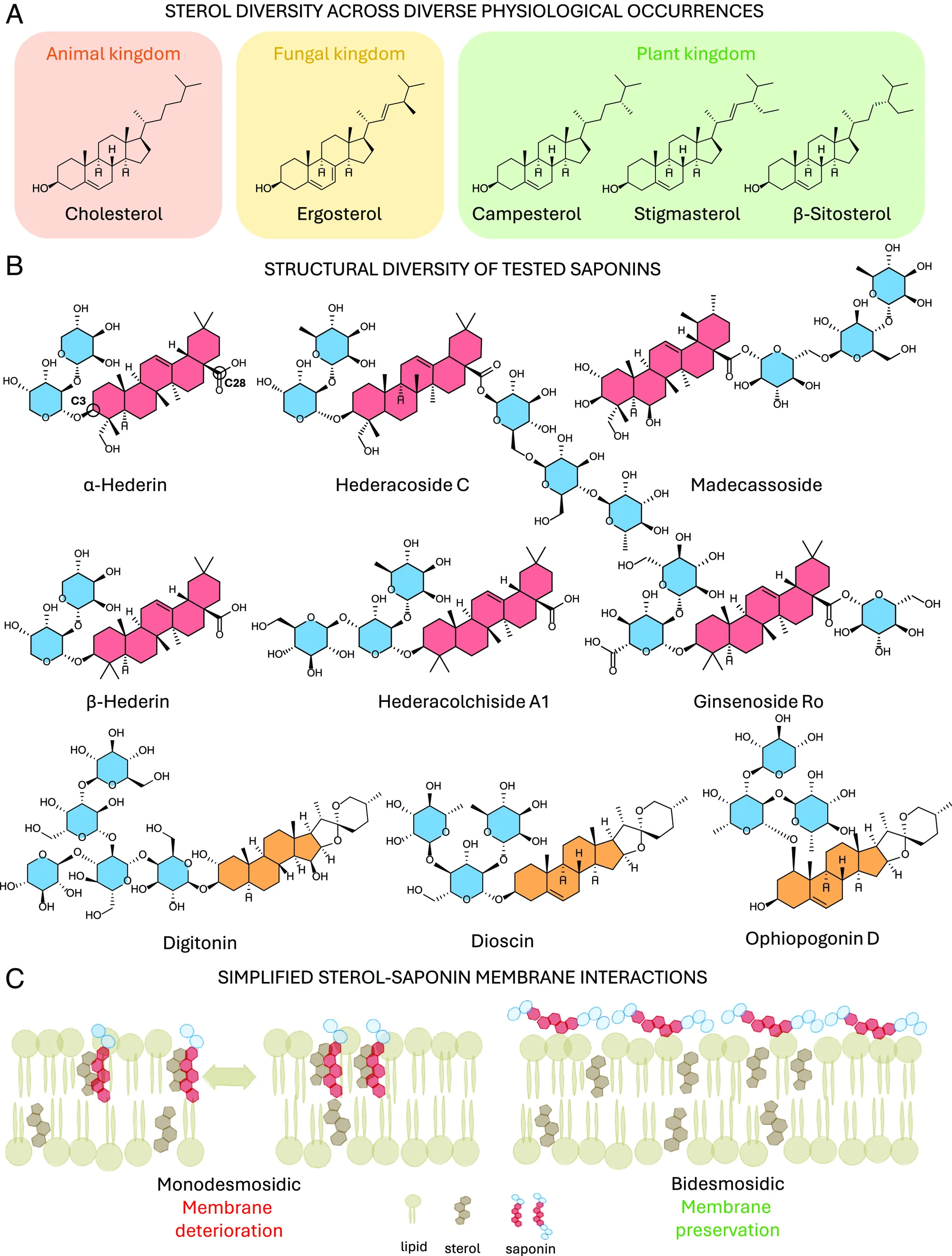
Sterol diversity and structural classification of saponins. (*A*) Chemical structures of representative sterols used within this study, categorized by their primary physiological occurrence: cholesterol, the predominant sterol in animals (orange); ergosterol, characteristic of fungi (yellow); and the phytosterols campesterol, stigmasterol, and β-sitosterol, found in plants (green). (*B*) Structures of the investigated saponins, grouped into triterpenoid (red backbone) and steroidal (orange backbone) classes. Hederagenin-derived saponins include the monodesmosidic α-hederin and bidesmosidic hederacoside C. Oleanolic type saponins include hederacolchiside A1, ginsenoside Ro, and β-hederin, as well as madecassoside. Finally, C28 glycosylated monodesmosidic madecassoside. Steroidal saponins include digitonin, dioscin, and ophiopogonin D. Monodesmosidic saponins carry a single sugar chain, typically at C3, whereas bidesmosidic saponins contain additional glycosylation at C28. (*C*) Schematic representation of saponin–membrane interactions. Monodesmosidic saponins exhibit stronger sterol-dependent membrane insertion and disruption, whereas bidesmosidic saponins show reduced membrane affinity.

Sterols are fundamental determinants of eukaryotic membrane structure and function (1). Although eukaryotes synthesize sterols from cyclization of the common precursor 2,3-oxidosqualene, plants, fungi, and animals have evolved distinct sterol classes, including cholesterol (CHOL) in animals, ergosterol (ERGO) in fungi, and phytosterols such as campesterol (CAMP), stigmasterol (STIG), and β-sitosterol (β-SITO) in plants (2) (Fig. 1*A*). In addition to membrane sterols, plants biosynthesize a vast diversity of specialized triterpenoid (C30) and steroidal (C27) saponins that originate from a sterol predisposition (3, 4). Saponins are a widespread (5–9) structurally diverse class of glycosylated plant defense compounds that protect against insect pests and pathogens (10–13). They consist of a hydrophobic triterpenoid or steroid aglycone (sapogenin) covalently linked to one or more hydrophilic sugar chains (14, 15) (Fig. 1*B*).

The selective mechanism by which saponins disintegrate cellular membranes is not fully understood, nor is the paradox of how plants avoid self-damage when biosynthesizing and storing saponins. Saponins are hypothesized to insert into membranes via their hydrophobic sapogenin, interact with membrane sterols, and form sterol–saponin complexes that alter lipid packing and membrane curvature, leading to pore formation, sterol sequestration, or disruption of lipid domains (9) (Fig. 1*C*). Saponin-induced membrane disruption may depend on interactions with CHOL or ERGO (16), however, the impact of sterol identity remains unclear.

We previously demonstrated the kingdom-specific bioactivity of triterpenoid saponins across four phylogenetically distinct organisms. The phytosterol-producing green algae *Raphidocelis subcapitata* exhibited lower susceptibility than organisms containing ERGO or CHOL, including *Daphnia magna*, *Enchytraeus crypticus*, and *Saccharomyces cerevisiae* (17). In this paper, to define how sterol composition across plants, animals, and fungi shapes saponin activity, we examined saponin–sterol interactions across these three kingdoms. We investigated a panel of nine structurally related saponins, including both monodesmosidic triterpenoid saponins such as α- and β-hederin, hederacolchiside A1, and madecassoside, as well as bidesmosidic triterpenoid saponins hederacoside C, and ginsenoside Ro. In addition, steroidal saponins such as digitonin, dioscin, and ophiopogonin D were included to capture structural variation in the aglycone scaffold and sterol-binding properties. The selected compounds also differ in the number and position of glycosylation sites, thereby enabling an assessment of how glycosylation patterns influence membrane interaction and lytic activity. Together, this panel enables a systematic comparison of how aglycone class, glycosylation pattern, and molecular interaction influence sterol-dependent membrane disruption. Using yeast mutants (18), synthetic membranes (16, 19), and molecular dynamics simulations (20–22), we integrate in vivo, in vitro, and in silico approaches to uncover how sterol identity and abundance modulate saponin-induced membrane disruption.

## Results

**Fig. 2.**
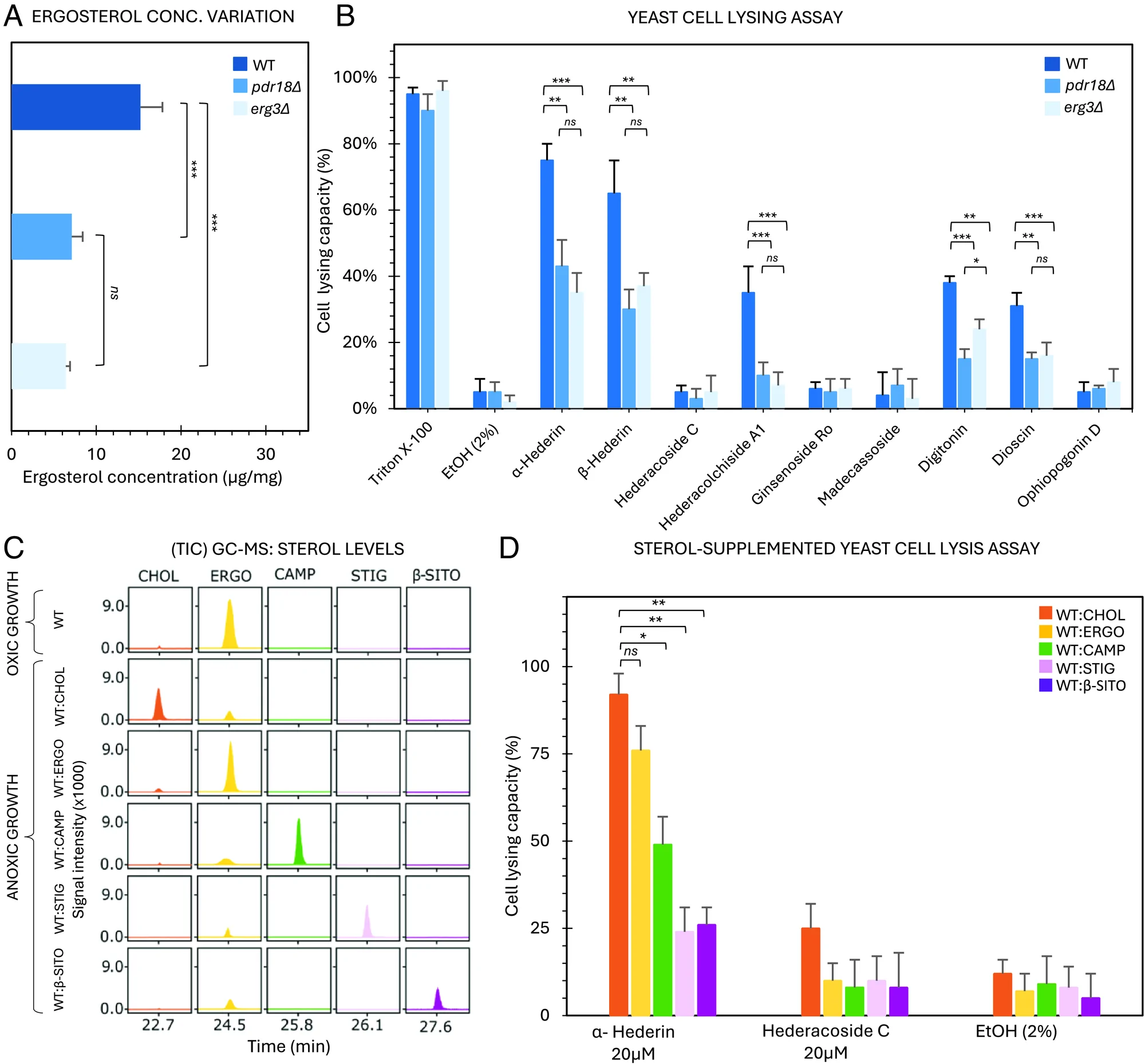
Sterol composition determines saponin activity in yeast membranes. (*A*) Measured ergosterol levels for the three strains of interest showing a significant decrease in ergosterol content of 45 to 65% in the mutant strains (*pdr18Δ* (middle, blue) and *erg3Δ* (*Bottom*, light blue)) compared to WT (*Top*, dark blue). (*B*) Cell lysis capacity (Nucleocounter assay) of saponins and controls in WT (dark blue), *pdr18Δ* (blue), and *erg3Δ* (light blue) strains. A general reduction in active saponin (α-hederin, -hederin, hederacolchiside A1, digitonin and dioscin) cell lysis is observed in mutant strains with lower ergosterol levels (n = 5). (*C*) GC-MS analysis of sterol composition in wild-type (WT) yeast and anaerobically grown yeast supplemented with exogenous sterols. A reduction in ergosterol (24.5 min, yellow) production is observed in anaerobically grown strains, while the supplemented sterol is incorporated (cholesterol (22.7 min, orange), campesterol (25.8 min, green), stigmasterol (26.1 min, pink), and β-sitosterol (27.6 min, purple) into the membrane. (*D*) Cell lysis capacity of saponins and controls in WT strains supplemented with exogenous sterols. A significant decrease in α-hederin activity is observed upon the introduction of phytosterols (n = 5). Significance was indicated as *P* < 0.05 (*), *P* < 0.01 (**), and *P* < 0.001 (***).

To assess the role of ERGO in modulating saponin bioactivity in yeast, strains with different ERGO levels (wild type (WT), *pdr18Δ* (impaired in transport of ERGO to membranes), and *erg3Δ* (impaired in ERGO biosynthesis)) were used. Absolute quantification confirmed a significant reduction of 40 to 65% in ERGO content in both mutant strains compared to WT (Fig. 2*A*). The yeast strains were treated with 20 μM of saponin, and cell viability was determined using propidium iodide staining. Cell lysis induced by the active saponins α-hederin, β-hederin, hederacolchiside A1, digitonin, and dioscin was approximately twofold higher in WT cells than in *pdr18Δ* and *erg3Δ* mutants, demonstrating that increased ERGO abundance correlates with increased susceptibility (Fig. 2*B* and Dataset S1). In contrast, the structurally different saponins hederacoside C, ginsenoside Ro, madecassoside, and ophiopogonin D caused no detectable lysis regardless of ERGO levels, indicating limited membrane-disruptive capacity.

Having established that ERGO abundance influences lytic susceptibility, we next tested whether sterol identity alone is sufficient to modulate saponin activity. To assess sterol-dependent effects on saponin bioactivity, yeast was cultured anaerobically in the presence of individual sterols (CHOL, ERGO, CAMP, STIG, or β-SITO) to enrich the yeast membrane with the respective sterol (Fig. 2*C*). Upon exposure to α-hederin, ERGO- and CHOL-supplemented cells showed high levels of lysis (~75% and 90%, respectively), mimicking the aerobic WT phenotype. Conversely, supplementation with plant-derived sterols conferred resistance to α-hederin: Lysis was reduced to 47% (CAMP), 23% (STIG), and 25% (β-SITO) (Fig. 2*D* and Dataset S1).

**Fig. 3.**
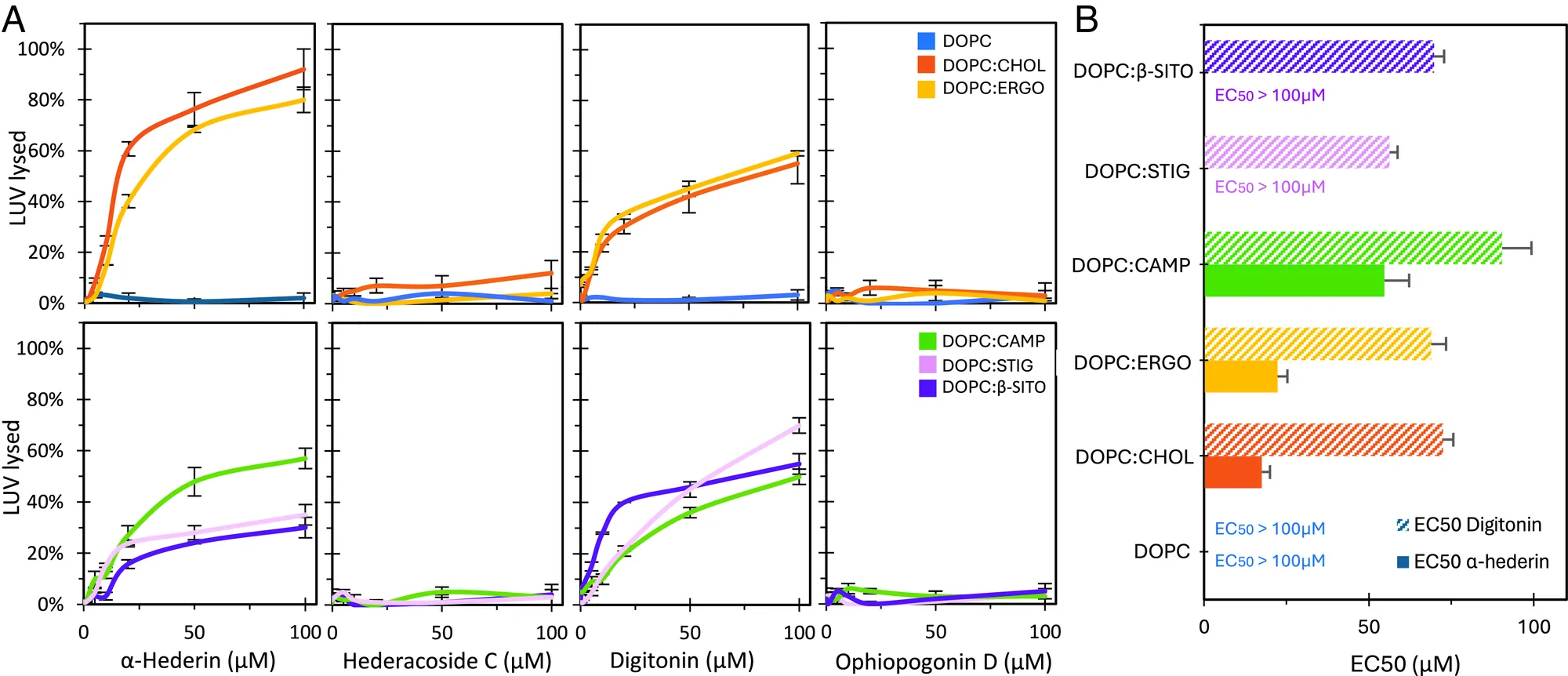
Sterol composition determines saponin-induced membrane disruption in model liposomes. (*A*) Saponin-induced lysis of Large Unilamellar Vesicles (LUVs) by α-hederin (first column), hederacoside C (second column), digitonin (third column), and ophiopogonin D (fourth column) in LUVs containing different sterols (n = 3, mean ± SD) divided in two groups, lipids with no sterol (blue), CHOL (orange), or ERGO (yellow) on the upper row and group with phytosterols CAMP (green), STIG (pink), and β-SITO (purple) lower row. (*B*) EC_50_ values of LUVs with different sterols with SD for both active saponin α-hederin (full) and digitonin (striped). No EC_50_ values were obtained for hederacoside C and ophiopogonin D, for DOPC-only LUVs with either α-hederin or digitonin, or for DOPC:STIG and DOPC:β-SITO LUVs when exposed to α-hederin.

To determine whether these sterol-dependent effects are intrinsic to membrane composition rather than concentration or yeast-specific factors, we used calcein-filled large unilamellar vesicles (LUVs) composed of dipalmitoylphosphatidylcholine (DOPC) and individual sterols in a 9:1 molar ratio and treated them with increasing concentrations of α-hederin, hederacoside C, digitonin, and ophiopogonin D. LUV membranes containing CHOL or ERGO exhibited the highest susceptibility to α-hederin–induced lysis (Fig. 3*A* and *SI Appendix*, Fig. S1), with EC_50_ values of 17.5 ± 2.5 µM and 22.4 ± 3.0 µM, respectively (Fig. 3*B* and Dataset S2). In contrast, phytosterol-containing LUVs were less susceptible to lysis: CAMP-containing LUVs showed an EC_50_ of 54.7 ± 7.3 µM, while STIG- and β-SITO-containing LUVs did not reach an EC_50_ within the tested concentration range (0 to 100 µM). The steroidal saponin digitonin showed intermediate EC_50_ values (between 49 and 99 µM; Fig. 3*B*) across all sterol-supplemented LUVs, indicating reduced sterol selectivity compared to α-hederin. LUVs lacking sterol did not lyse, confirming that sterols are required for saponin-induced lysis. An upper concentration of 100 µM was chosen to remain within physiologically relevant ranges and to avoid saponin precipitation (23).

**Fig. 4.**
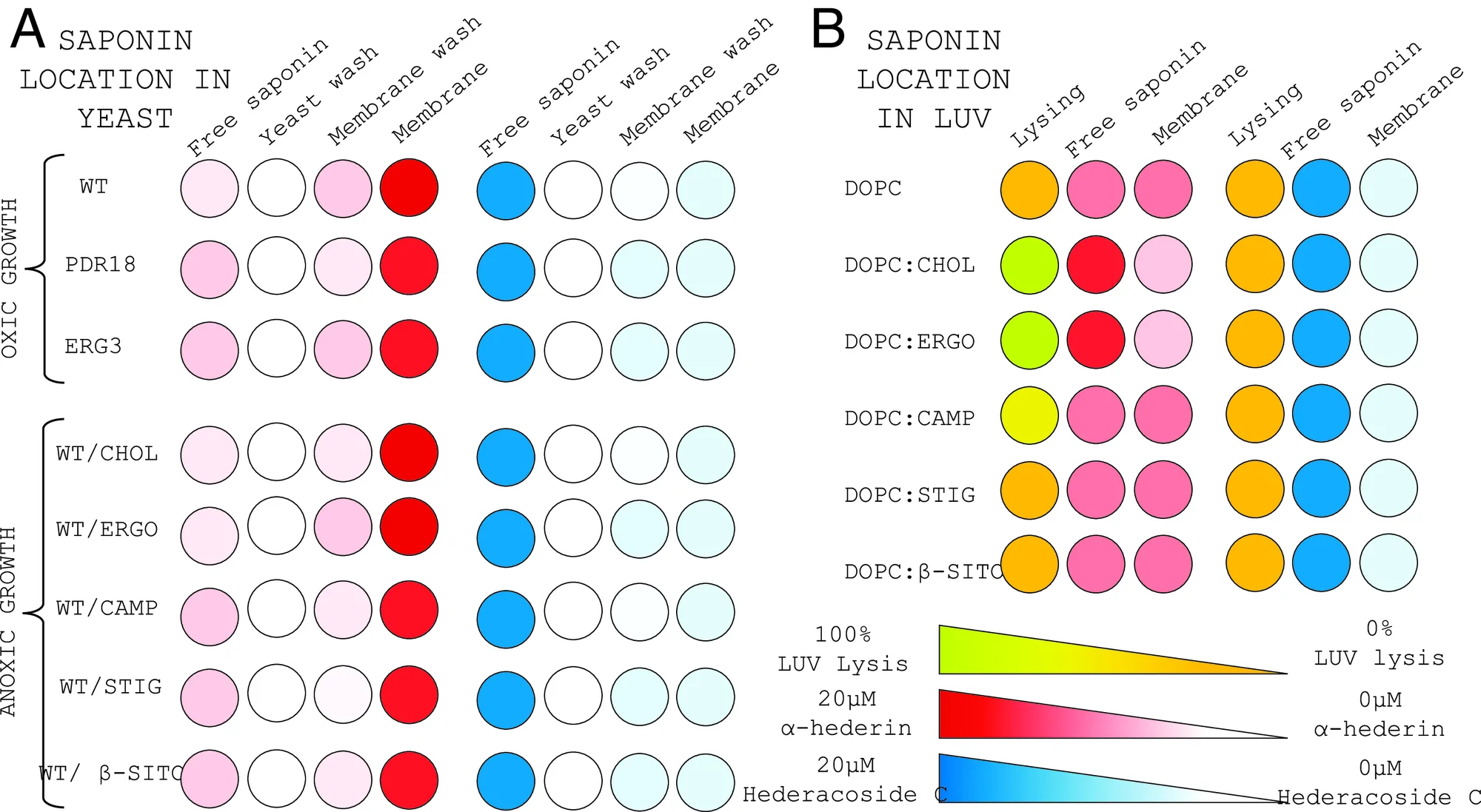
Distinct membrane partitioning of monodesmosidic and bidesmosidic saponins. (*A*) Relative localization of saponins α-hederin (red, *Left*) and hederacoside C (blue, *Right*) across collected fractions: free saponin (supernatant), yeast wash, membrane wash, and membrane (membrane-associated saponins) for the yeast assay. (*B*) Lysis levels after saponin exposure, together with free saponin and membrane fractions for LUV. Hederacoside C is predominantly found in its free form for both yeast and LUV assays, suggesting minimal membrane interaction, while α-hederin is enriched in the membrane fraction, indicating potential sterol association.

To investigate whether sterol-dependent lysis reflects differences in membrane association, we analyzed the distribution of a representative mono- and bidesmosidic saponin in membrane and supernatant fractions using differential centrifugation. Monodesmosidic α-hederin predominantly accumulated in the membrane fraction of ERGO- and CHOL-enriched yeast cells (Fig. 4*A*). When yeast was grown anaerobically to facilitate phytosterol incorporation, the signal of membrane-associated α-hederin was slightly reduced. As anticipated, the bidesmosidic hederacoside C was detected predominantly in the supernatant and showed little membrane association. To substantiate these findings, the experiment was repeated using sterol-supplemented LUVs (Fig. 4*B*). In LUV systems, α-hederin was detected in both membrane and supernatant fractions; however, CHOL- and ERGO-containing LUVs showed detectable membrane-bound α-hederin, whereas phytosterol-containing LUVs exhibited reduced membrane association. A substantial fraction of α-hederin remained in the supernatant, likely reflecting vesicle lysis and soluble membrane fragments being too small for pelleting at 100,000 x g. In contrast, hederacoside C was consistently detected in the supernatant across all sterol compositions, consistent with its minimal lytic activity.

**Fig. 5.**
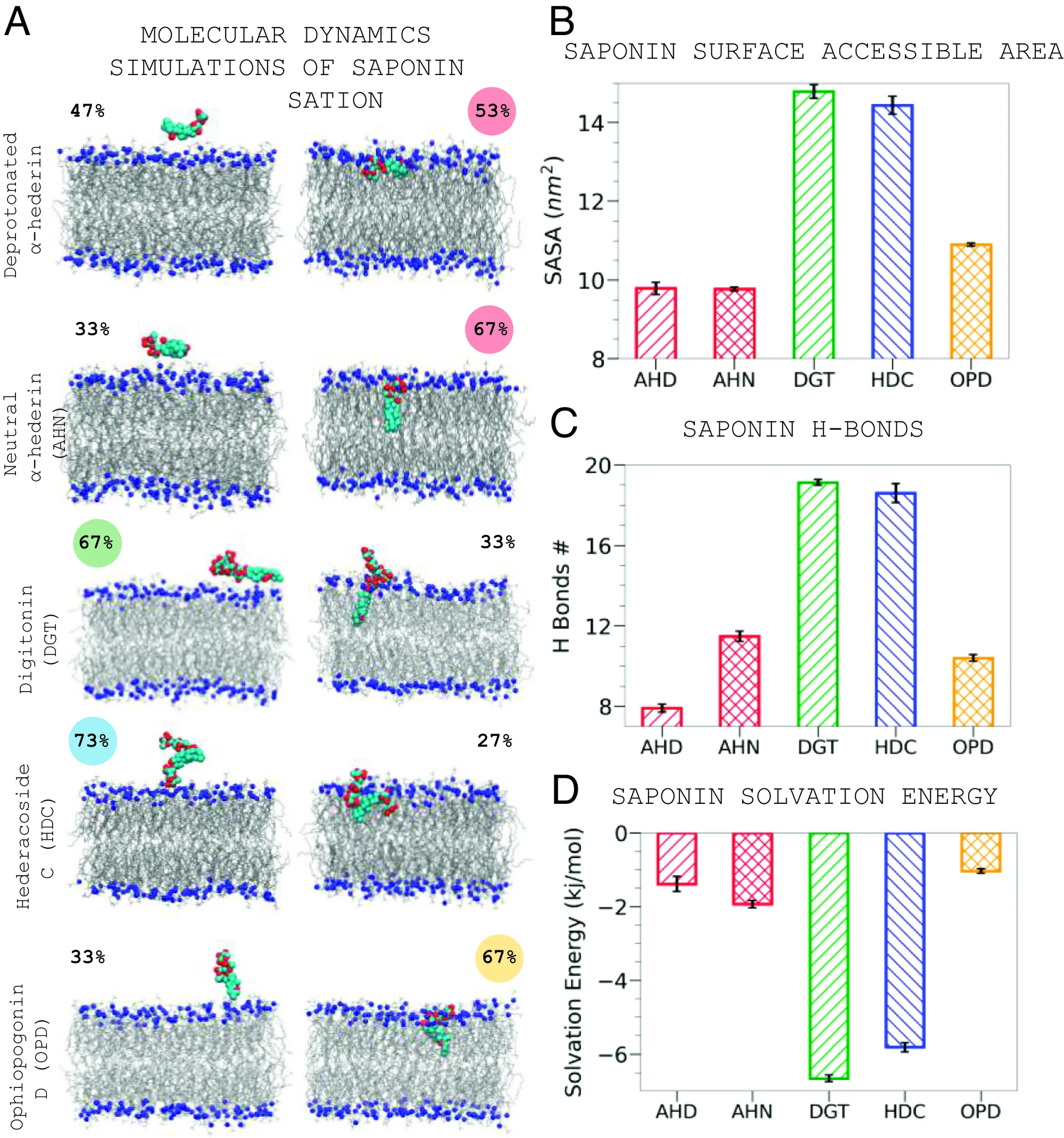
Biophysical determinants of saponin–membrane interactions. (*A*) Representative bound and unbound configurations for deprotonated α-hederin (AHD, red), neutral α-hederin (AHN, red), hederacoside C (HDC, blue), digitonin (DGT, green), and ophiopogonin D (OPD, yellow). Percentages indicate the proportion of systems in which the corresponding event was observed relative to the total number of simulated systems (also summarized in *SI Appendix*, Table S3). Left side correlates with the percentage not interacting with the membrane while the right side corresponds with the percentage interacting with the membrane. (*B*) Solvent-accessible surface area (SASA), (*C*) number of hydrogen bonds with water, and (*D*) solvation energy for AHD, AHN, DGT, HDC, and OPD. All measurements were obtained from saponin-in-water simulation systems run in triplicate, the error bars represent the SE across the replicas.

To determine whether intrinsic physicochemical properties explain differences in membrane activity, we performed molecular dynamics (MD) simulations of the monodesmosidic saponin α-hederin and the structurally related bidesmosidic saponin hederacoside C in aqueous solution and in model membranes. Because α-hederin contains a free carboxyl group at C28, both its protonated (neutral) and deprotonated (charged) forms were simulated. In aqueous solution, hederacoside C exhibited a larger solvent-accessible surface area (SASA) compared to α-hederin (Fig. 5*B*), indicating that a greater fraction of its molecular surface remains exposed to water. Consistent with this, hederacoside C formed more hydrogen bonds with surrounding water molecules (Fig. 5*C*) and displayed a more favorable solvation energy (Fig. 5*D*), reflecting stronger hydration and an increased preference for the aqueous phase.

In the saponin–membrane simulations, α-hederin is rapidly associated with the bilayer. Both protonated and deprotonated forms are inserted into the membrane, although with distinct orientations and insertion depths. Quantitatively, approximately 67% of protonated α-hederin molecules and 53% of the deprotonated form remained stably membrane-inserted over the simulation time, whereas only ~27% of hederacoside C molecules exhibited stable membrane association (Fig. 5*A* and *SI Appendix*, Table S3). The majority of hederacoside C molecules remained in the aqueous phase.

To further assess membrane retention, hederacoside C was artificially positioned within the bilayer at simulation onset. In these simulations, hederacoside C gradually dissociated from the membrane and returned to the aqueous phase, whereas α-hederin remained membrane-associated under comparable conditions (Movies S1 and S2). Together, these results demonstrate that the monodesmosidic α-hederin possesses intrinsic membrane affinity, while the additional sugar chain in hederacoside C increases hydration and disfavors stable bilayer insertion.

To determine whether saponin structural class further modulates membrane engagement, we compared the triterpenoid saponin α-hederin with the steroidal saponins digitonin and ophiopogonin D. While intrinsic membrane affinity explained differences between α-hederin and hederacoside C, the relationship between membrane association and lytic activity among steroidal saponins was less straightforward. MD simulations of single saponin-bilayer systems showed that digitonin exhibited ~33% membrane association, whereas ophiopogonin D displayed a higher interaction frequency (~67%) (Fig. 3*A* and *SI Appendix*, Table S3). However, this predicted interaction frequency did not directly correlate with experimental lytic activity: Digitonin induced measurable lysis in vivo and in vitro, whereas ophiopogonin D showed minimal lysis. These findings indicate that simple membrane association frequency alone is insufficient to explain lytic potency.

To explore whether insertion geometry influences membrane-disruptive capacity, we examined the conformational organization of the two steroidal saponins in membrane-bound states. Structural modeling revealed that ophiopogonin D adopts a folded or bent conformation, with an angle of approximately 60 to 135° between the aglycone and sugar moieties, whereas digitonin assumes a more extended configuration (~180°) (*SI Appendix*, Fig. S5*D*). In representative membrane-bound configurations, digitonin adopts a predominantly vertical insertion mode, with the aglycone penetrating deeper into the bilayer core, while ophiopogonin D remains more surface-oriented due to its folded architecture. This distinction in insertion geometry provides a mechanistic explanation for the discrepancy between membrane association frequency and lytic activity. Although ophiopogonin D can associate with the bilayer, its folded conformation likely limits effective hydrophobic contact with the membrane core and does not promote the membrane reorganization associated with lysis. In contrast, the more linear configuration of digitonin permits deeper insertion and is consistent with its measurable lytic activity.

Simulations performed at higher saponin concentrations further indicated that cooperative effects influence membrane engagement. Multisaponin simulations revealed that, among the four simulated saponins, only digitonin was capable of spontaneous insertion without forming persistent aqueous aggregates (*SI Appendix*, Fig. S2 and Table S4), indicating differences in cooperative insertion behavior between structural classes and supporting the measurable membrane-binding ability of digitonin under experimentally relevant, nondilute conditions.

To determine how sterol composition modulates membrane susceptibility under multimolecule conditions, we simulated systems containing five saponins initially embedded in DOPC:sterol bilayers (200:5 lipid:saponin ratio). These simulations were designed to capture cooperative membrane behavior and quantify how sterol identity influences membrane organization following saponin insertion.

**Fig. 6.**
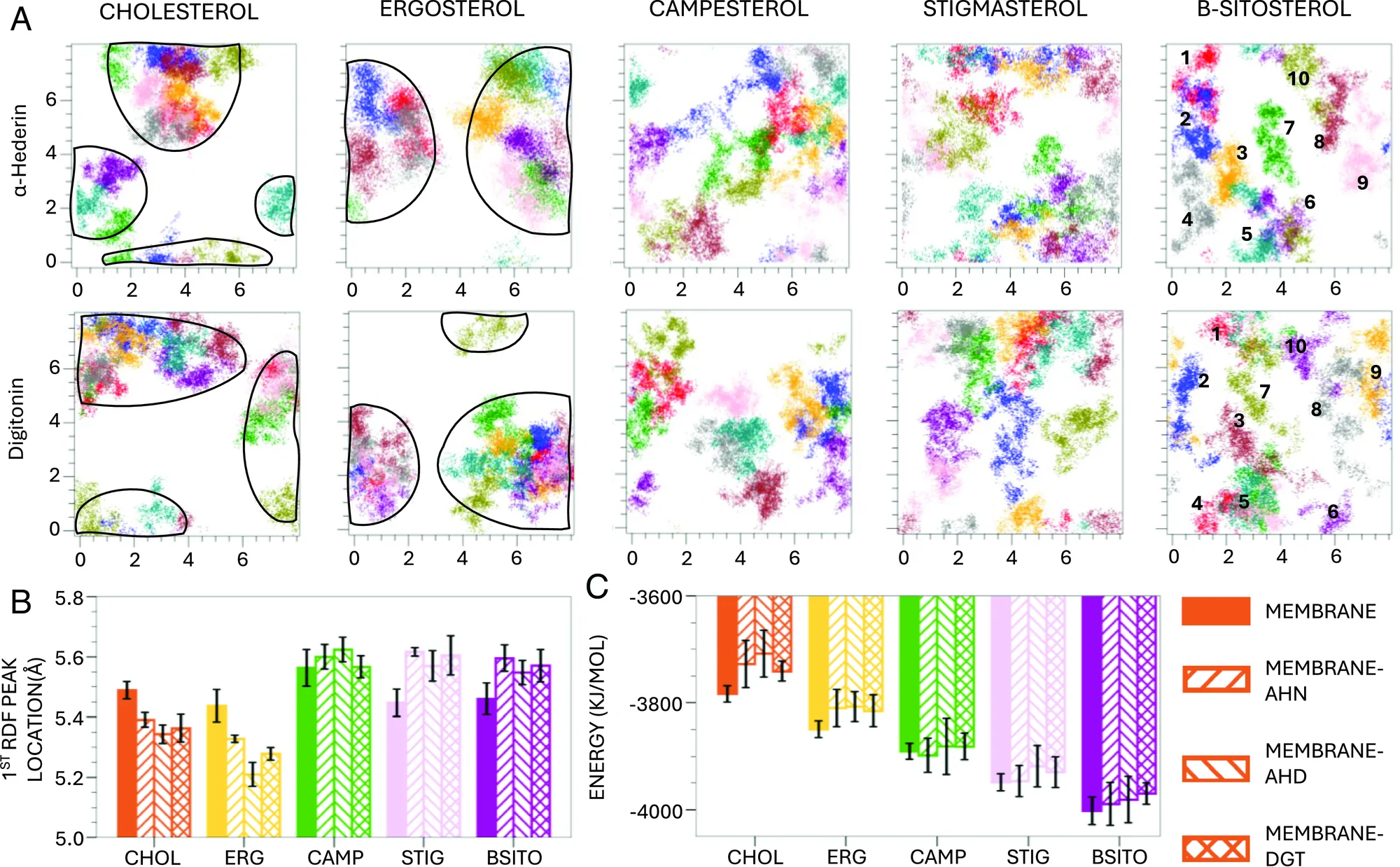
Molecular modeling of membrane–saponin interaction provides mechanistic insights into saponin bioactivity. (*A*) Cumulative sterol distribution maps from the last 100 ns of simulations for membranes with five bound deprotonated α-hederin molecules (*Top* row) and digitonin molecules (*Bottom* row). Colorful scatter points in the distribution map represent the positions of the individual sterol hydroxyl oxygen atoms. Individual cholesterol molecules are labeled in the last column as reference. A higher aggregation of the animal and fungi sterols (black line) compared to the phytosterols is observed. (*B*) Position of the 1st peak in 2D radial distribution function (RDFs) for sterol hydroxyl oxygen atoms in membrane-only simulation systems (solid bars) and membranes containing five neutral (AHN, left-diagonal filled bars), five deprotonated α-hederin molecules (AHD, right-diagonal filled bars) and five digitonin molecules (DGT, cross filled bars). (*C*) Corresponding nonbonded interaction potential energies between DOPC and sterols.

Cumulative sterol distribution maps show that CHOL and ERGO exhibit enhanced local clustering when exposed to saponins, whereas phytosterols (CAMP, STIG, and β-SITO) remain more evenly dispersed with limited redistribution (Fig. 6*A*). These observations were quantified using two-dimensional radial distribution functions (RDFs) describing lateral sterol–sterol organization based on in-plane distances between sterol hydroxyl oxygen atoms. In CHOL- and ERGO-containing membranes, insertion of α-hederin induced a leftward shift in the first RDF peak, indicating closer sterol–sterol packing. Phytosterol-containing membranes exhibited minimal RDF shifts or, in some cases, rightward shifts, consistent with reduced sterol clustering (Fig. 6*B* and *SI Appendix*, Fig. S3). The direction and magnitude of these sterol reorganization patterns parallel experimental lysis trends. Membranes that display increased sterol clustering in silico (CHOL and ERGO) are also the most susceptible to α-hederin–induced lysis in vivo and in vitro, whereas phytosterol-containing membranes show both limited clustering and reduced lytic susceptibility.

To explore the physicochemical basis for these differences, we calculated nonbonded interaction energies between DOPC and individual sterol species. CHOL and ERGO exhibited weaker interactions with DOPC compared to phytosterols (Fig. 6 *B* and *C*). These weaker lipid–sterol interactions are consistent with a greater propensity for sterol redistribution upon saponin insertion, whereas stronger DOPC–phytosterol interactions correlate with increased structural stability and reduced sterol reorganization. Digitonin-containing systems also altered sterol organization, but with reduced sterol selectivity compared to α-hederin. Two-dimensional RDF analysis revealed leftward shifts for CHOL and ERGO, whereas phytosterols generally exhibited minimal changes relative to membrane-only systems. This more modest sterol-specific response is consistent with digitonin’s intermediate lytic potency and comparatively limited sterol selectivity observed experimentally.

In contrast, the inactive steroidal saponin ophiopogonin D did not promote ERGO clustering. In DOPC/ERGO bilayers containing five ophiopogonin D molecules, sterol distribution maps and RDF analyses indicated no enhancement of sterol aggregation relative to the membrane-only condition. This lack of sterol reorganization is consistent with its minimal lytic activity in vivo and in vitro (*SI Appendix*, Fig. S5 *A*–*C*).

Together, these findings demonstrate that saponin architecture governs membrane disruption through coordinated control of intrinsic membrane affinity, insertion geometry, and cooperative membrane engagement. Importantly, membrane association alone does not predict lytic activity. Instead, productive insertion and sterol reorganization emerge as key determinants of sterol-dependent susceptibility.

## Discussion

Plant saponins are widely recognized as membrane-active defense metabolites, yet the determinants of their specificity remain incompletely understood. By examining nine plant-derived saponins spanning triterpenoid and steroidal subclasses across in vivo, in vitro, and in silico systems, we identify key structural and sterol determinants that govern membrane lysis. We demonstrate that membrane sterols are required for efficient saponin-induced lysis and that sterol identity determines susceptibility: ERGO and CHOL enhance lysis, whereas phytosterols confer resistance. These results indicate that plant saponin-based chemical defense systems are closely aligned with the biophysical properties of membrane sterols, with variation in membrane sterol composition strongly influencing lytic activity.

Manipulation of sterol composition in yeast provided direct causal evidence for this model. Mutants impaired in ERGO homeostasis have previously been reported to exhibit increased resistance to several bioactive saponin compounds (24). In alignment, reduced ERGO levels in our system (*erg3Δ* and *pdr18Δ*) consistently decreased susceptibility to membrane-lytic saponins, indicating that ERGO availability is a key determinant of membrane vulnerability. This suggests that previous discrepancies in sterol mutant phenotypes may reflect compound-specific differences in sterol dependence rather than a general resistance mechanism. Complementary sterol replacement experiments under anaerobic conditions further demonstrated that susceptibility is determined by sterol identity: Supplementation with ERGO or CHOL restored sensitivity to α-hederin, whereas phytosterols (CAMP, STIG, and β-SITO) reduced lytic activity. Similar sterol-dependent protection has been reported for the triterpenoid saponin escin, which is derived from the horse chestnut (*Aesculus hippocastanum*), where ERGO supplementation in media confers a protective characteristic for the exposed yeast strains (25, 26). Together, these findings indicate that sterols actively modulate the efficiency of membrane destabilization rather than merely permitting saponin interaction.

Our data further reveals that intrinsic structural features of saponins determine their sterol-dependent activity. Monodesmosidic triterpenoid saponins exhibited the highest lytic potency, whereas bidesmosidic saponins displayed minimal membrane disruption across systems, consistent with reduced membrane affinity. The glycosylation pattern impacts activity: Increased glycosylation reduced activity, likely by increasing solvent hydration and limiting membrane insertion, the reduced bioactivity of hederacolchiside A1 relative to α- and β-hederin supports this model. Steroidal saponins showed broader but generally weaker sterol selectivity, although digitonin had activity across sterol environments. These observations suggest that pentacyclic triterpenoid aglycones provide a more effective biophysical affinity for rigid fungal and animal sterols, maximizing hydrogen bonding interactions with ERGO and CHOL membranes.

Importantly, the sterol-dependent selectivity observed in yeast was substantiated in LUVs. LUV membranes containing ERGO or CHOL were most susceptible to lysis by α-hederin, whereas phytosterols reduced membrane lysis (27). Sterol-deficient membranes were largely resistant, confirming that sterols are required for efficient lysis (16). These findings indicate that sterol identity directly shapes membrane susceptibility through intrinsic lipid–sterol interactions. However, previous studies have demonstrated that steroidal saponins such as digitonin and dioscin exhibit pronounced membrane-disruptive activity in sterol-containing systems, with a strong affinity for ERGO- and CHOL-rich membranes (28, 29). Structure–activity analyses of steroidal saponins, including tigogenin- and diosgenin-derived compounds, showed that glycosylation pattern and aglycone scaffold are critical determinants of antifungal potency (30). These observations indicate that both triterpenoid and steroidal saponins lyse sterol-containing membranes, yet their distinct structural features differentially modulate membrane disruption, highlighting the potential impact of a given saponin structure to achieve tailored selectivity for specific sterol compositions.

Molecular dynamics simulations provided mechanistic insight into these sterol-dependent effects. Both α-hederin and digitonin induced sterol redistribution and lateral clustering in CHOL- and ERGO-containing membranes, consistent with enhanced susceptibility. Such reorganization was minimal in phytosterol-containing membranes, supporting the hypothesis that sterol identity governs membrane response to saponin insertion. Similar sterol clustering and domain formation have been reported for α-hederin in cholesterol-rich bilayers (31), and adaptive sterol modification (cholesterol to Δ7-sterols) has been observed in organisms such as sea cucumbers, which alter membrane sterol composition to reduce susceptibility to endogenous saponins (32). These parallels suggest that sterol diversification represents a conserved biological strategy to modulate membrane vulnerability.

The simulations also clarified intrinsic determinants of membrane engagement. Monodesmosidic triterpenoid saponin, α-hederin, is readily inserted into bilayers in both neutral and deprotonated states, consistent with accessibility of the hydrophobic aglycone. Protonation of the C-28 carboxyl group may enhance insertion depth by increasing effective hydrophobicity, as suggested by recent studies (33). In contrast, bidesmosidic saponins exhibited limited membrane insertion due to steric hindrance and increased hydration (34, 35). Computational analysis of solvent-accessible surface area (SASA), hydrogen bonding with water, and solvation energy demonstrated that additional sugar moieties increase hydration and favor retention in the aqueous phase. These findings are consistent with the proposed role of bidesmosidic saponins as inactive storage forms that can be enzymatically converted to more active monodesmosidic forms upon tissue damage, generating a rapid chemical defense response (36).

**Fig. 7.**
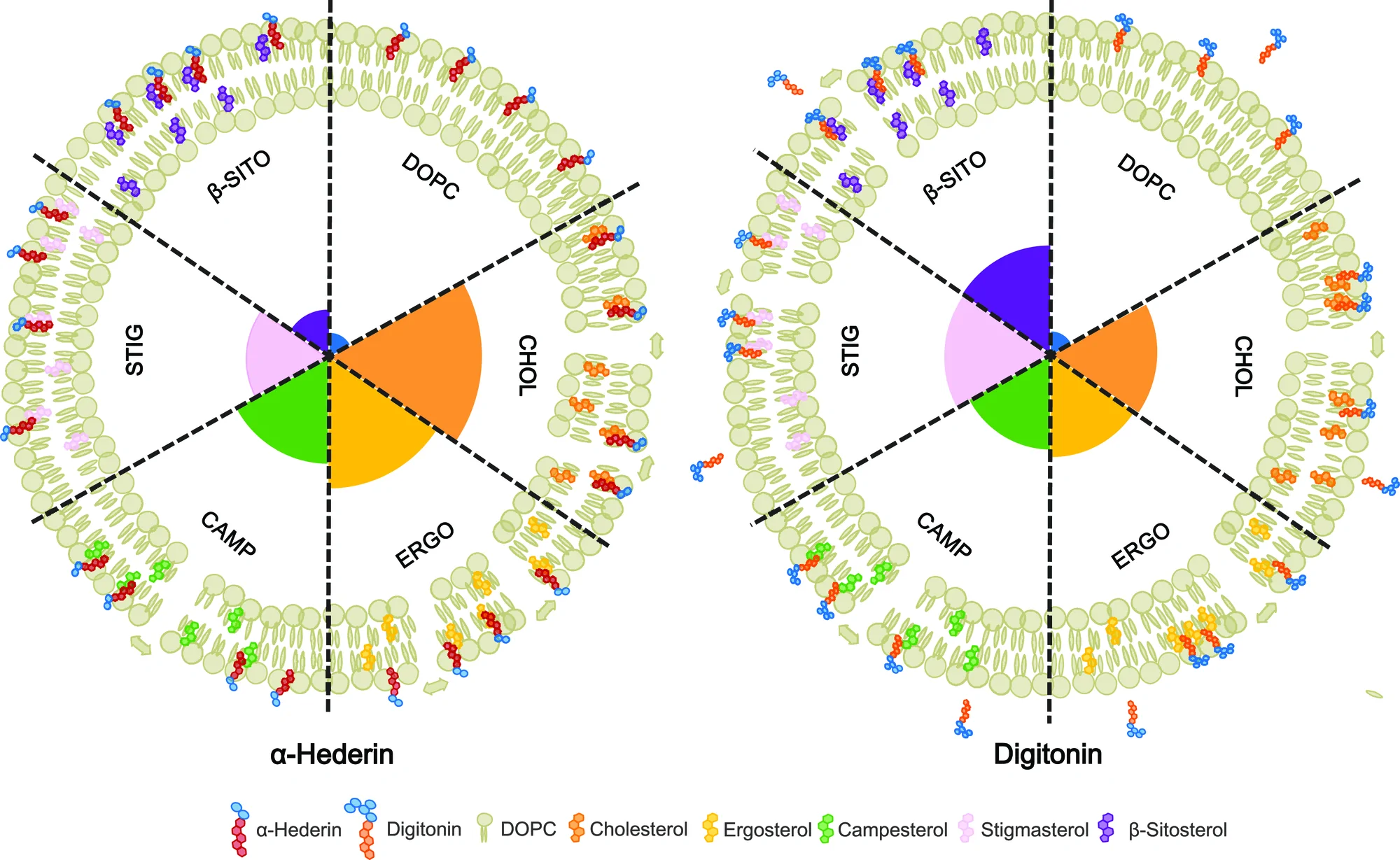
A new two-parameter model of saponin–membrane interactions. Saponin-induced membrane disruption is governed by two key determinants: i) the molecular structure of the saponin, which defines its aggregation, membrane affinity, and insertion behavior, and ii) the sterol composition of the target membrane, which modulates susceptibility to lysis. The contrasting interaction profiles of the triterpenoid α-hederin and the steroidal digitonin illustrate this dual control. The sector area represents relative lytic activity (larger = stronger effect); colors indicate sterol type. α-Hederin shows strong and selective activity toward ergosterol- and cholesterol-containing membranes, whereas digitonin exhibits weaker and less discriminatory interactions across sterol compositions.

Together, the in silico analyses support a hierarchical model in which i) intrinsic membrane affinity is determined by saponin glycosylation state, ii) insertion geometry differs between triterpenoid and steroidal subclasses, and iii) sterol identity dictates the extent of membrane reorganization following insertion. This multilevel compatibility between saponin structure and sterol composition (Fig. 7) provides a mechanistic framework for understanding sterol-dependent susceptibility observed in vivo and in vitro. This compatibility model also resolves a long-standing paradox in plant biology: Plants accumulate high concentrations of amphiphilic, membrane-active saponins without compromising their own membranes. Our findings suggest that phytosterol-rich membranes inherently resist sterol redistribution and destabilization, providing intrinsic protection against endogenous saponins. In contrast, fungal and animal membranes containing ergosterol or cholesterol are more permissive to sterol clustering and membrane disruption. This coevolution between saponin biosynthesis and membrane sterol composition therefore represents a coordinated chemical–biophysical defense strategy.

To our knowledge, this study provides the first unified cross-kingdom model linking saponin structure, sterol identity, and membrane reorganization to explain selective saponin lysis across plants, animals, and fungi. Rather than acting as indiscriminate surfactants, saponins emerge as sterol-selective membrane modulators whose activity reflects evolutionary compatibility between small-molecule structure and membrane composition. This framework not only refines our understanding of plant chemical defense but also offers a predictive basis for designing saponin-based antifungals, adjuvants, and membrane-active agents with improved selectivity. More broadly, it highlights sterol diversification as an evolutionary strategy for resistance to a prevalent class of membrane-active natural products.

## Materials and Methods

Detailed experimental procedures are described in the *SI Appendix, Materials and Methods* for all experimental setups.

### Yeast Viability Assay.

Membrane permeabilization in yeast was quantified using a propidium iodide (PI)-based viability assay. PI uptake was used to assess plasma membrane integrity following saponin treatment. Fluorescence-based cell analysis enabled quantitative comparison of sterol-dependent sensitivity across strains (37–39).

### Yeast Membrane–Saponin Interaction Assay.

To assess sterol-dependent saponin partitioning in cells, yeast strains with defined sterol compositions were exposed to saponins followed by subcellular fractionation. Differential centrifugation was used to separate extracellular, cell-associated, and membrane-enriched fractions. The resulting fractions (free saponins, yeast wash, membrane wash, and membrane fraction) were analyzed by LC–MS to quantify sterol-dependent interactions (40).

### Liposome Preparation.

Large unilamellar vesicles (LUVs) with defined sterol compositions were prepared as described previously (41). LUV permeabilization was quantified using a calcein leakage assay, in which dye release upon saponin treatment reports membrane disruption.

### Saponin-Induced Calcein Release Assay.

Calcein-loaded LUVs were exposed to increasing saponin concentrations, and membrane lysis was quantified by fluorescence-based detection of calcein release. Signals were normalized to complete vesicle lysis to enable quantitative comparison across sterol compositions. Release (%) was calculated using a dilution-corrected formula (1), adapted from Claereboudt et al. (42):

[1] $$I\left(\%\right)=\left(\frac{I_{1}-0.89\left(I_{0}\right)}{I_{2}-0.89\left(I_{0}\right)}\right)*100,$$

Where I_0_, I_1_, and I_2_ are fluorescence before saponin, after saponin, and after Triton X-100, respectively. A dilution factor of 0.89 was introduced to recalculate the initial intensity before dilution.

### Liposome Separation and Saponin Localization.

LUVs (500 μL) were incubated with 20 μM saponin for 15 min at room temperature (~22 °C), followed by centrifugation at 100,000× *g* for 60 min (Optima MAX-XP, Beckman Coulter) to separate intact membranes from unbound saponin. Fractions were analyzed by LC–MS for saponin content.

### Sterol Analysis by GC-MS.

Wild-type and mutant *S. cerevisiae* strains (WT, *erg3Δ*, *pdr18Δ*) were used to generate different membrane sterol compositions under aerobic and anaerobic growth conditions. Cellular sterols were extracted, saponified, derivatized, identified, and quantified by internal standard–based GC–MS (43, 44). Analyses were performed on a Shimadzu GC-MS Nexis-2030 platform equipped with a capillary column (30 m × 0.25 mm × 0.25 μm, Agilent).

### Saponin Analysis by LC–MS.

Saponins were detected and quantified by UHPLC–qTOF–MS using a reversed-phase C18 column with a water–acetonitrile gradient containing formic acid, as previously described (45). Analyses were performed on a Dionex UltiMate 3000 UHPLC system coupled to a Bruker Compact qTOF-MS with electrospray ionization operating in negative ion mode (full-scan and MS/MS) with internal calibration.

### Molecular Dynamics Simulations: Bilayer Setup and Saponin 3D Models.

Symmetric DOPC:sterol (9:1) bilayers were constructed to match the lipid composition of the LUV systems (46–48). Sterols include CHOL, ERGO, ERGO, β-SITO, and STIG. Membrane-only simulations were conducted to characterize pure membrane properties and served as controls. Structures of α-hederin (protonated and deprotonated), hederacoside C, digitonin, and ophiopogonin D were generated and parameterized using the AVOGADRO and CHARMM General Force Field (49–52), respectively. Simulations of saponin-in-water systems were performed to equilibrate saponin structures and calculate their properties. All simulations were conducted in triplicate to ensure reproducibility, and results are reported as averages ± SE (*SI Appendix*, Table S1).

### Saponin–Membrane Systems Setup Simulation.

Equilibrated saponin and membrane coordinates were combined to investigate aggregation, membrane insertion, and sterol-dependent membrane responses. Systems were neutralized with KCl ions. Three initial configurations were simulated: i) a single saponin positioned 10 to 15 Å above the membrane; ii) five saponins positioned 10 to 15 Å above the membrane; and iii) five saponins preinserted into the bilayer. For inserted systems, starting configurations were generated by pulling saponins from the aqueous phase toward the membrane hydrophobic region using a harmonic bias (53, 54) implemented in PLUMED (55–57). Systems were subsequently equilibrated with positional restraints at the membrane center, followed by unbiased production simulations to characterize saponin–membrane interactions (*SI Appendix*, Table S2).

### Simulation Settings.

All saponin–membrane systems were simulated using the CHARMM36m force field and TIP3P water model (58–60). Independent replica simulations were performed to ensure reproducibility and quantify statistical uncertainty, yielding a total of 74 μs of sampling (*SI Appendix*, Table S3). Simulations were conducted under physiological temperature and pressure conditions using GROMACS 2021.5 (61–63). Long-range electrostatics and van der Waals interactions were treated using established molecular dynamics approaches, and the LINCS constraint algorithms were applied (64–68).

### Statistical Analysis.

Group differences were assessed using independent *t* tests or one-way ANOVA, as appropriate. Significant ANOVAs were followed by post hoc tests with corrections. Normality and homogeneity of variances were verified prior to analysis. Significance was indicated as *P* < 0.05 (*), *P* < 0.01 (**), and *P* < 0.001 (***).

### EC_50_ Determination.

Dose–response data were analyzed for LUV experiments in R studio (version. 2022.02.3, 2022) using nonlinear regression. A four-parameter logistic (4PL) model was fitted with the *drc* package, and EC_50_ values were estimated using the Levenberg–Marquardt algorithm. EC_50_ and SE were obtained directly from the model output, and 95% CI were calculated by profile likelihood. Initial EC_50_ estimates based on concentrations up to 100 μM were likely underestimated, as the response of some curves did not reach a clear upper plateau. Extending the concentration range enabled full sigmoidal fitting and more accurate estimation of both E_max_ and EC_50_.

### AI Use Disclaimer.

AI tools, including ChatGPT (GPT-3.5, OpenAI), were used to improve clarity, grammar, and structure of the manuscript during initial drafting and revision stages. AI was not used for idea generation, data analysis, scientific interpretation, or reference retrieval. All scientific content, analyses, and conclusions were developed and verified by the authors.

## Supplementary Material

Appendix 01 (PDF)

Dataset S01 (XLSX)

Dataset S02 (XLSX)

Movie S1. **Interaction of α-hederin and hederacoside C with lipid membranes.** Compared with the DOPC bilayer (left), the monodesmosidic protonated saponin α-hederin penetrates more deeply into the membrane in the presence of ergosterol (center). In contrast, the bidesmosidic saponin hederacoside C does not sufficiently penetrate the membrane core and instead remains on the surface of the lipid bilayer (right).

Movie S2. **Interaction of saponins with DOPC/CHOL bilayers under multi-molecule conditions.** Neutral α-hederin (left), deprotonated α-hederin (center), and hederacoside C (right) were initially pulled into the membrane by an external force during the first 5 s of the movie (corresponding to the first 20 ns of the simulation trajectory). The force was then removed, allowing spontaneous saponin-membrane interactions. Neutral α-hederin penetrates deepest into the membrane, whereas hederacoside C remains near the membrane surface and may dissociate into the water phase, consistent with the higher water affinity of bidesmosidic saponins.

## Data Availability

Study data are included in the article and/or supporting information. Simulation data have been deposited in Zenodo (69).
