# Supplementary material for: Sterol divergence across eukaryotic kingdoms determines membrane susceptibility to saponins, a class of plant defense compounds
Source: Proc Natl Acad Sci U S A. 2026 May 8;123(19):e2523859123. doi: 10.1073/pnas.2523859123 (PMC13168540; doi:10.1073/pnas.2523859123)
Supplement: Supplementary file 1 — Appendix 01 (PDF) [file pnas.2523859123.sapp.pdf]

PNAS

Supporting Information for

Sterol divergence across eukaryotic kingdoms determines membrane susceptibility to saponins, a class of plant defense compounds

Malbor Dervishi[1], Jan Günther[1], Jinhui Li[2], Huriye Deniz Uzun[1,3], Hans Christian Bruun Hansen[1], Thomas Günther Pomorski[1,3], Anja Thoe Fuglsang[1], Viviana Monje[2], and Søren Bak[1,*]

Department of Plant and Environmental Sciences, University of Copenhagen, Thorvaldsensvej 40, 1871 Frederiksberg, Denmark[1]
Department of Chemical and Biological Engineering, University of Buffalo, 308 Furnas Hall, Amherst, NY 14260, United States[2]
Department of Molecular Biochemistry, Faculty of Chemistry and Biochemistry, Ruhr University Bochum, 44780 Bochum, Germany[3]

* Corresponding author (Søren Bak)
E-mail address: bak@plen.ku.dk

This PDF file includes:

Supporting text (detailed material and method)
Figures S1 to S5
Tables S1 to S4
Legends for Movies S1 to S2
Legends for Datasets S1 to S2
SI References

Other supporting materials for this manuscript include the following:

Movies S1 to S2
Datasets S1 to S2

Supporting Information Text

Detailed Materials and Methods

Chemicals

The following chemicals were used in the study: α-hederin (Extrasynthesis, France); hederacoside C Analytical standard (Sigma-Aldrich, Germany); propodium iodide (Thermofisher, Austria); 3-(N-morpholino)propanesulfonic acid, MOPS (Sigma-Aldrich, Denmark); polyoxyethylene sorbitan monolaurate, TWEEN® 20 (Roche, Germany); ergosterol (ERGO, >95%), cholesterol (CHOL, >99%), campesterol (CAMP, >65%; ~35% dihydrobrassicasterol), stigmasterol STIG >99%, β-sitosterol (β-SITO, >99%), all purchased through Sigma-Aldrich (Germany); 1,2-di-(9Z-octadecenoyl)-sn-glycero-3-phosphocholine, DOPC (Avanti, USA); Sephadex G-50 (Sigma-Aldrich, USA); bis[N,N−bis(carboxymethyl)aminomethyl]fluorescein, calcein (Sigma-Aldrich, USA); N,O-bis(trimethylsilyl)trifluoroacetamide, BSTFA (Sigma-Aldrich, Switzerland).

Yeast Strains and growth

All strains were purchased from Euroscarf (Germany): Wild type BY4741 (MATa *his3Δ1 leu2Δ0 met15Δ0 ura3Δ0*); *erg3Δ* (BY4741; MATa *his3Δ1; leu2Δ0; met15Δ0; ura3Δ0;* YLR056w::kanMX4) and *pdr18Δ* (BY4741; MATa; *his3Δ1; leu2Δ0; met15Δ0; ura3Δ0*; YNR070w::kanMX4).

Yeast strains were cultured overnight at 28 °C and 150 rpm (Innova 44, New Brunswick Scientific, USA) in 20 mL YPD medium supplemented with 20 µg/L carbenicillin, starting at an OD600 of 0.2 in 100 mL Erlenmeyer flasks. Cultures typically reached an OD600 of ~10. For each assay, 1 mL of culture was transferred to a 2 mL Eppendorf tube and washed three times with buffer A (100 µM MOPS; 1 mM KCl; 2% w/v glucose; pH 7, adjusted with Trizma base (Sigma-Aldrich, Denmark). Washing was performed by centrifugation at 14,000 rpm for 2 minutes (Centrifuge 5424, Eppendorf, Germany) to separate the yeast pellet from the supernatant. After the final wash, the pellet was resuspended in buffer A to an OD600 of 0.2[1,2]. Anaerobic growth was performed under the same conditions using a Microbiology Anaerocult A Mini kit (Millipore, Germany), with 40 µg/L sterol and 0.6% v/v Tween 20 added to the medium[3]. Similar to aerobic growth, each test was carried out by collecting an individual population, and OD600 measurements were taken before and after incubation to confirm normal growth.

Yeast viability assay

Yeast cells were grown overnight in YPD medium, diluted to an OD600 of 0.2 in fresh medium, incubated at 28 °C for 3 hours to reach the logarithmic growth phase. Cells were then washed three times with buffer A and resuspended in the same buffer to the original OD600. For treatment, 49 µL of the yeast suspension was mixed with 49 µL of propidium iodide (PI) (3 µg/ml dissolved in buffer A) and 2 µL of the test compound[4] (saponin 20 µM, Triton X-100 or EtOH >96%) in PCR tubes. After 30 minutes of incubation at 28 °C with shaking (150 rpm), 30 µL of each sample was loaded into a chamber of an NC-Slide A2 (100 µm, Chemometec, Denmark). Membrane integrity was assessed using the NucleoCounter NC-3000 (Chemometec, Denmark), which detects PI-bound DNA, indicating membrane permeabilization (39). Instrument settings were as follows: green channel (excitation 475 nm, emission 560–535 nm), dark field masking; exposure time: 1000; emission filter: Em 675/75; adaptive parameters: maximum number of analyzed cells, and exclusion of aggregates enabled.

Yeast membrane–Saponin interaction assay

Yeast strains (WT, *erg3Δ*, *pdr18Δ*) were grown aerobically or anaerobically cultured with 40 µg/L sterol and 0.6 % (v/v) Tween 20, as highlighted. After 16 hours, membranes were isolated for LC-MS analysis of saponin interaction. Cultures (1 mL) were washed three times with buffer A, adjusted to OD600 = 0.2 in 980 µL buffer A, and exposed (in triplicate) to 20 µL α-hederin or hederacoside C (1 mg/mL) for 15 min. Cells were centrifuged at 10,000 × g (Eppendorf 5424),

yielding pellet and supernatant (free saponins). Pellet was resuspended in 5% EtOH, sonicated for 60 min (Branson 5510), and centrifuged again at 10,000 × g to yield yeast wash and supernatant. Supernatant was ultracentrifuged at 100,000 × g (Beckman Optima MAX-XP) to isolate the membrane fraction from the final supernatant membrane wash. Free saponin, membrane fraction, and both yeast and membrane wash were analyzed by LC-MS.

Liposome preparation

Large unilamellar vesicles (LUVs) were prepared by extrusion essentially as described (23). Briefly, 5 mg DOPC and sterol (9:1 molar ratio) were dissolved in chloroform, mixed, and subjected to three cycles of chloroform evaporation using a rotary evaporator (Rotovap RII, Buchi, Switzerland) to form a uniform dry lipid film. The film was rehydrated with 500 µL loading buffer (62.75 mg calcein in 1 mL buffer A, pH-adjusted with 1 N NaOH for a final calcein concentration of 100mM) and glass beads (212–300 µm), then vortexed for 10 min at maximum intensity (3.200 rpm; Vortex-Genie-2, Sigma-Aldrich). Following five freeze–thaw cycles (10 min in liquid nitrogen/ 5 min in 50 °C water bath), the suspension was extruded 11 times through a 0.2 µm filter using a 1 mL syringe. LUVs were purified by passing the sample twice through a Sephadex G-50 column to remove unencapsulated calcein as described[5].

Saponin-Induced Calcein Release Assay

Calcein-loaded LUVs were diluted 1:10 in buffer A, and 180 µL was added per well of a black 96-well plate (Thermo Scientific, Denmark). Saponin (diluted in EtOH) was added (20 µL) to achieve final concentrations of 0–100 µM; EtOH served as a solvent control. After 15 min incubation, 4 µL Triton X-100 (20% (w/v) Triton-X-100) was added to lyse vesicles and determine total calcein release. Fluorescence (Ex/Em: 495/515 nm) was recorded in triplicate at three time points at 25°C room temperature: baseline (180 µL), post-saponin (200 µL), and post-lysis (204 µL). Release (%) was calculated using a dilution-corrected formula (1), adapted from Claereboudt et al. [6]:

$$I(\%) = \left(\frac{I_1 - 0.89(I_0)}{I_2 - 0.89(I_0)}\right) * 100 \quad (1)$$

Where I0, I1, and I2 are fluorescence before saponin, after saponin, and after Triton X-100, respectively. A dilution factor of 0.89 was introduced to recalculate the initial intensity before dilution.

Liposome separation and saponin localization

LUVs (500 µL) were incubated with 20 µM saponin for 15 min at room temperature (~22 °C). The mixture was then ultracentrifuged at 100,000 × g for 60 min (Optima MAX-XP, Beckman Coulter) to separate intact membranes from unbound saponin. Both pellets, membrane fraction (membrane-associated) and supernatant (free saponin) fractions were analyzed by LC-MS.

Sterol analysis by GC-MS

For sterol analysis, yeast strains (WT, *erg3Δ, pdr18Δ*) were grown aerobically; WT was also cultured anaerobically with 40 µg/L sterol and 0.6 % (v/v) Tween 20. Cultures (1 ml) were adjusted to OD600 = 5.0 before being pelleted and washed three times with buffer A supplemented with 2% (v/v) glucose, then lysed in 1 mL 60% w/v KOH solution containing 10 µM 5α-cholestane (internal standard, >95%, Sigma-Aldrich). Samples were vortexed and incubated at 90°C for 2 hours[7]. Sterols were extracted three times with hexane, pooled, and evaporated at 55°C under nitrogen flow. Samples were subsequently redissolved in 100 µL hexane; 30 µL was mixed with 30 µL BSTFA (Sigma-Aldrich) and derivatized at 60 °C for 1 h.

Derivatized samples were analyzed by GC-MS (Shimadzu GCMS Nexis-2030) using an HP-5MS UI capillary column (30 m × 0.25 mm × 0.25 µm, Agilent). Helium was used as the carrier gas (30 cm/s). A 2 µL sample was injected in splitless mode at 250 °C. The GC temperature program: 60 °C (1 min), ramp to 280 °C over 7 min, then to 310 °C over 36 min, followed by re-equilibration at 60 °C[8].

Saponin analysis by LC-MS

Saponins were analyzed on a Dionex UltiMate 3000 UHPLC system (Thermo Fisher Scientific, Germany) coupled to a Bruker Compact qToF-MS with ESI source (Bruker Daltonics, Germany). Separation was achieved on a Kinetex 1.7 µm XB-C18 column using a mobile phase of 0.05% formic acid in water (A) and acetonitrile with 0.05% formic acid (B). The gradient was as follows: 0.0–1.0 min with 5% B; 1.0–2.0 min from 5–30% B; 2.0–14.0 min from 30–70% B; 14.0–15.0 min at 70–100% B; 15.0–16.0 min at 100% B; 16.0–17.0 min back to 5% B; and 17.0–20.0 min at 5% B. Flow rate was 300 µL/min, column temperature was 30°C[9]. MS conditions were as follows: ESI in negative mode, spray voltage −3900 V, dry gas (N2) flow 8 L/min at 250 °C, nebulizer pressure 2.5 bar, collision energy 10 eV. MS data were collected over m/z 50–1400; MS/MS from m/z 200–1400 at 3 Hz. Ammonium formate was used for internal calibration via Data Analysis 4.3 (Bruker) as described[10]. Samples were filtered through 0.2 µm Durapore membranes (Millipore) and stored at 4 °C prior to analysis.

Molecular dynamics simulations: Bilayer setup and Saponin 3D models

Symmetric DOPC:sterol (9:1) bilayers (referred to as membrane-only systems) were built to match the lipid compositions of LUVs using the CHARMM-GUI Membrane Builder[11-13]. Sterols in this study include CHOL, ERGO, CAMP, β-SITO, and STIG. Each bilayer was fully hydrated and neutralized with a 0.15 mol/L KCl ion solution. Membrane-only systems were relaxed using the 6-step CHARMM-GUI protocol for bilayers, followed by a 50 ns production run prior to the insertion of saponin structures[14]. In addition, each membrane-only system was further simulated for an additional 250 ns to characterize the properties of different sterol membranes, which served as controls for the saponin-membrane systems.

Initial 3D coordinates of α-hederin (in protonated or deprotonated states) and hederacoside C were constructed and geometrically optimized using Avogadro[15]. The structure was parametrized using the CHARMM General Force Field (CGenFF)[16,17] and solvated using CHARMM-GUI Solution Builder[18,19]. Each saponin system (referred to as saponin-in-water systems) was relaxed using the default minimization and equilibration protocols from CHARMM-GUI Solution Builder. Subsequently, a 150 ns trajectory was run to equilibrate the structure before merging its coordinates in the membrane-only systems. The final 100 ns was used for structural analysis of saponins. All the above membrane-only and saponin-in-water systems were built and run in triplicate to ensure reproducibility and report averages and standard errors accordingly (Table S1).

Saponin-membrane systems setup

Equilibrated coordinates of the saponins and membranes were merged to examine saponin aggregation, membrane insertion mechanisms, and associated membrane response according to sterols. As before, neutralizing KCl ions were used on each saponin-membrane system to render the simulation box neutral. The simulation of the following starting scenarios: (i) a single saponin molecules positioned 10-15 Å above the membrane; (ii) five saponins of the same type positioned 10-15 Å above the membrane; (iii) five saponin molecules inserted into the membrane. The starting configurations for the third scenario were generated by pulling saponin molecules from the water phase toward membrane tail region using a constant harmonic bias (KAPPA = 10 kJ/mol/nm2 for individual saponins) via the MOVINGRESTRAINT function[20,21] in PLUMED/2.7.141-43. A 10 ns equilibration trajectory was then performed with individual saponins harmonically restrained at the membrane center, defined by the center of mass of the lipid phosphorus atoms in both leaflets. The restraints were finally removed prior to running the unbiased trajectories to examine saponin-membrane interactions (Table S2).

Simulation settings

All saponin-membrane systems were simulated using the Charmm36m force field[22,23] and TIP3P water model[24]. As with membrane-only and saponin-in-water systems, each saponin-membrane system simulation was built and run in triplicate to ensure reproducibility and quantify statistical uncertainty. Each replica was simulated for 500 ns, for a total of 42.5 µs of simulation data (Table S3). NPT ensemble at 303.5K and 1 bar using the Nose-Hoover thermostat[25] and Parinello-Rahman barostat[26] in GROMACS/2021.5 software package[27] was utilized. The LINCS algorithm

was used to constrain bonds involving hydrogen atoms during the trajectory[28]. Particle Mesh Ewald with a 1.2 nm cutoff was used to evaluate electrostatics[29], and the van der Waals potential with a force-switching function between 1-1.2nm to account for long-range interactions[30]. Unless otherwise specified, all remaining simulation parameters followed the default settings in GROMACS. Visual Molecular Dynamics (VMD) package[31] was used to visualize the systems and render snapshots. Trajectory analysis was conducted on VMD, GROMACS, and using the MD Analysis python library [32].

Statistical analysis

Group differences were assessed using independent t-tests or one-way ANOVA, as appropriate. Significant ANOVAs were followed by post hoc tests with corrections. Normality and homogeneity of variances were verified prior to analysis. Significance was indicated as $p < 0.05$ (*), $p < 0.01$ (**), and $p < 0.001$ (***).

EC50 Determination

Dose-response data were analyzed using nonlinear regression in R. A four-parameter logistic model (4PL) was fitted using the dose response modeling function from the drc package. The estimated EC50 (half-maximal effective concentration) was fitted using the experimental data using the Levenberg–Marquardt algorithm for parameter estimation. The EC50 value and standard error were extracted directly from the model output using summary and ED functions. Confidence intervals were calculated at 95% using profile likelihood methods. Although an initial EC50 value was estimated using concentrations up to 100 µM, the response curve of DOPC:CAMP did not reach a clear plateau (Figure S1; Data S2). This likely resulted in an underestimation of EC50, since the full dose-response relationship was not captured. By extending the concentration range, a more complete sigmoidal curve was obtained, allowing for a more accurate estimation of both the maximum effect (Emax) and the true EC50.

‘’

Figures

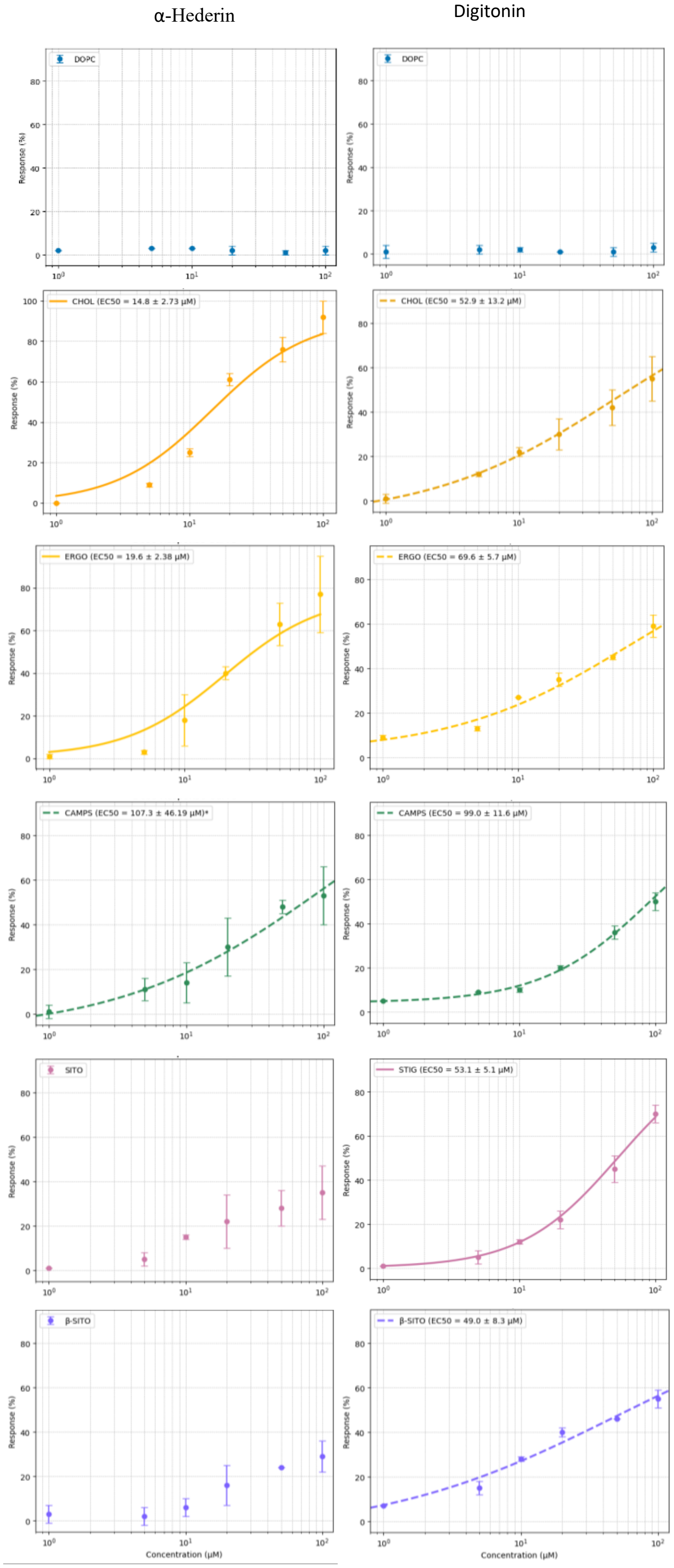

α-Hederin
Digitonin
DOPC
CHOL (EC50 = 14.8 ± 2.73 μM)
CHOL (EC50 = 52.9 ± 13.2 μM)
ERGO (EC50 = 19.6 ± 2.38 μM)
ERGO (EC50 = 69.6 ± 5.7 μM)
CAMPS (EC50 = 107.3 ± 46.19 μM)*
CAMPS (EC50 = 99.0 ± 11.6 μM)
SITO
STIG (EC50 = 53.1 ± 5.1 μM)
β-SITO
β-SITO (EC50 = 49.0 ± 8.3 μM)
Response (%)
Concentration (μM)

Figure S1. Sterol-dependent membrane lysis dose–response curves. Dose–response relationships for LUV lysis induced by different sterols (CHOL, ERGO, CAMPS, STIG, β-SITO) compared to DOPC control. Solid lines indicate fits within the experimentally supported response range (plateau reached or well-defined). Dashed lines indicate model-based extrapolations where the maximal response was not fully reached within the tested concentration range. Panels without a fitted curve represent datasets where no reliable sigmoidal behavior or EC50 could be determined (data shown without model fitting). Error bars represent standard deviation (SD). Concentrations are plotted on a logarithmic scale.

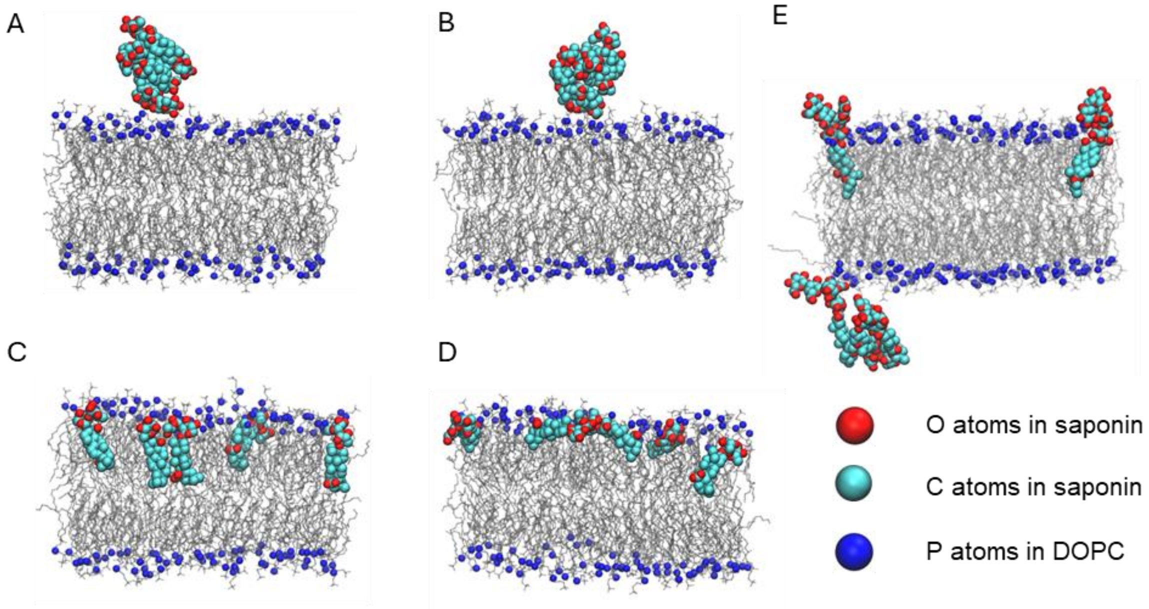

Figure S2: Final stages of the simulation systems with five α-hederin molecules initially located in the water phase near the bilayer for the (A) protonated and (B) deprotonated form and five α-hederin molecules initially in an embedded conformation in the DOPC/CHOL bilayer model for the (C) neutral and (D) deprotonated forms. Unlike other systems, five digitonin molecules can spontaneously insert the bilayer even when initially in water, without forming large aggregates like other saponin molecules do in water (E).

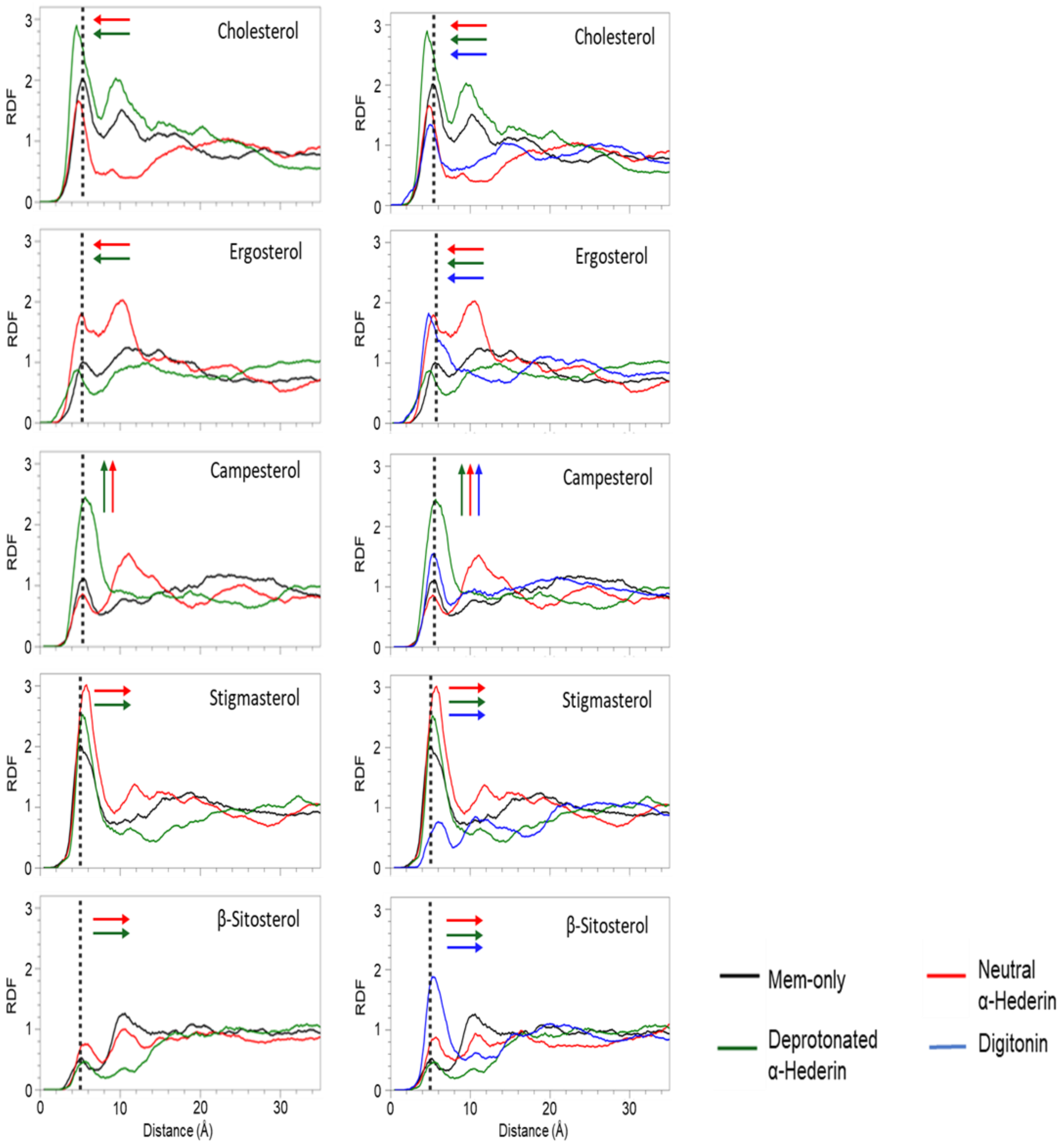

Figure S3: Two-dimensional radial distribution functions (RDFs) of sterol–sterol interactions. The curves represent the probability distribution of finding sterol molecules near each other, calculated based on the lateral (x–y plane) distance between their hydroxyl oxygen atoms. Black, red, and green lines correspond to membrane-only systems, membranes containing five neutral α-hederin molecules, membranes with five deprotonated α-

hederin molecules, and membranes containing five digitonin molecules, respectively. Vertical grey dashed lines indicate the position of the first solvation shell in membrane-only systems (i.e., without saponins).

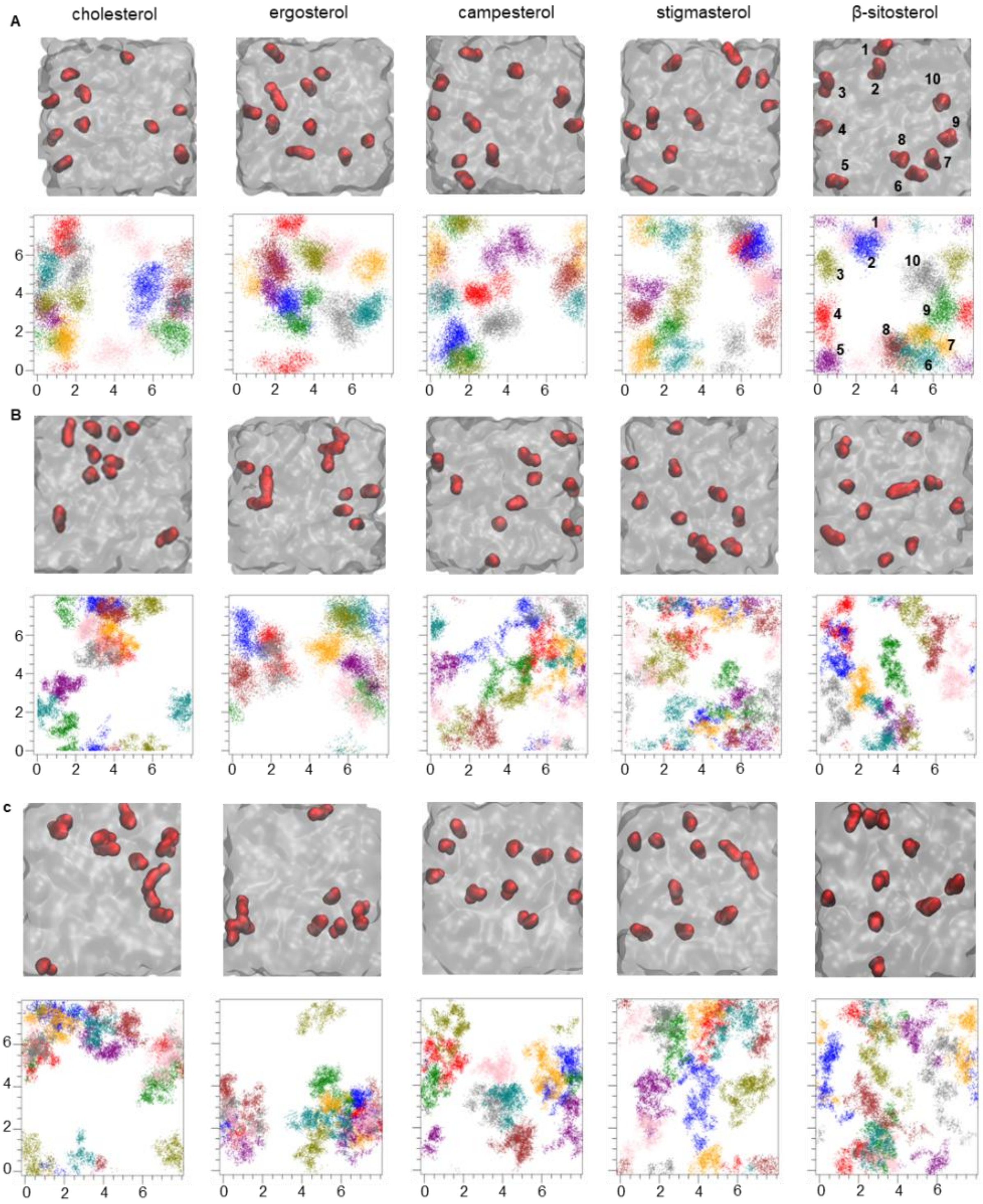

Figure S4: Molecular modeling of membrane-saponin interaction provides mechanistic insights into saponin bioactivity. Representative top-view snapshots and corresponding cumulative sterol distribution maps from the last 100 ns of simulations for (A) membranes-only systems, (B) membranes with five bound deprotonated α-hederin molecules, and (C) membranes with five bound digitonin molecules. Red globes represent sterols in the α-hederin-bound leaflet; grey represents DOPC lipids; and colorful scatter points in the distribution map

represent the positions of the individual sterol hydroxyl oxygen atoms. Individual cholesterol molecules are numbered in the last column as reference. We observe a higher natural aggregation of the sterols compared to the phytosterols. The aggregation of CHOL and ERGO increases with the presence of saponins.

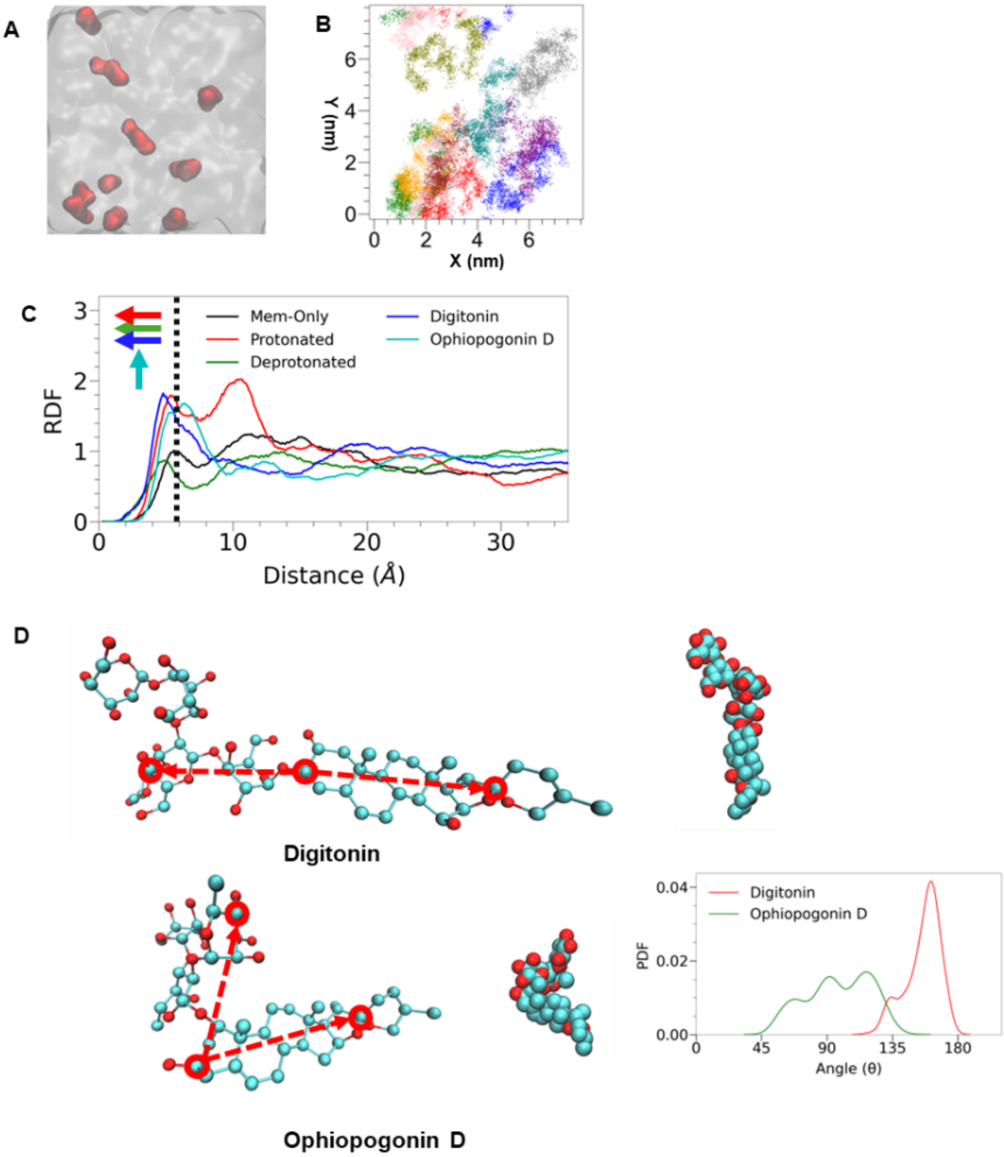

Figure S5. Ophiopogonin D does not promote ergosterol clustering in a DOPC/ERGO bilayer. Five ophiopogonin D molecules were initially placed below the phosphate groups of one leaflet of a DOPC/ERGO bilayer. Simulations were run for 500 ns, with the last 100 ns used for analysis. (A) Representative snapshot of the system; sterols are shown in red spheres, while grey surfaces represent DOPC lipids. (B) Lateral distribution maps of sterol hydroxyl oxygen atoms accumulated over the final 100 ns. Each colored scatter point represents the position of an individual sterol hydroxyl oxygen atom projected onto the membrane plane. (C) Two-dimensional radial distribution functions (RDFs) describing sterol–sterol lateral organization. RDFs were calculated based on the in-plane (x–y) distances between sterol hydroxyl oxygen atoms. (D) Representative structures and dihedral angle definition used to characterize molecular flexibility. Digitonin adopts a predominantly extended conformation (~180°), whereas ophiopogonin D displays a more compact and flexible structure. Probability density functions of the dihedral angle (θ) from molecular dynamics simulations highlight these distinct conformational preferences. Unlike digitonin, ophiopogonin D does not promote ergosterol aggregation, likely due to its folded conformation, which reduces the effective hydrophobic contact between the aglycone moiety and the bilayer hydrophobic core, its folded conformation, which reduces the effective hydrophobic contact between the aglycone moiety and the bilayer hydrophobic core.

Tables

Table S1. Summary of saponin-in-water and DOPC/sterol membrane-only systems.

System Names	Lipids /leaflet	Total atoms #	Simulation box dimensions (nm)	Sim. time × reps (ns)
**Saponin-in-water**				
Neutral α-Hederin	-	6989	4.11*4.11*4.11	150x3
Deprotonated α-Hederin	-	6983	4.11*4.11*4.11	150x3
Hederacoside C	-	6969	4.11*4.11*4.11	150×3
Digitonin	-	9037	4.47*4.47*4.47	150×3
Ophiopogonin D	-	6400	3.99*3.99*3.99	150×3
**Pure DOPC/sterol membranes**				
DOPC/CHOL	100	62433	7.78*7.78*9.93	300x3
DOPC/ERGO	100	62353	7.99*7.99*9.44	30ox3
DOPC/CAMP	100	62478	7.88*7.88*9.73	300x3
DOPC/STIG	100	62444	8.04*8.04*9.33	300x3
DOPC/ β-SITO	100	62529	7.91*7.91*9.92	300x3

Table S2. Summary of simulated systems with protonated α-Hederin (AHD), deprotonated α-Hederin (AHN), hederacoside C (HDC), digitonin (DGT), and ophiopogonin D (OPD), respectively.

Saponin	Sterol	Lipids /leaflet	Total atoms #	Simulation box dimensions (nm)	Sim. time × reps (ns)
**Single saponin-membrane (saponin starts from water)**					
AHD	CHOL	100	59823	7.91*7.91*9.22	500x3
	ERGO	100	59623	7.83*7.83*9.33	500x3
	CAMP	100	59883	7.82*7.82*9.41	500x3
	STIG	100	59323	7.74*7.74*9.54	500x3
	β-SITO	100	60050	7.89*7.89*9.26	500x3
AHN	CHOL	100	59817	7.76*7.76*9.52	500x3
	ERGO	100	59605	7.96*7.96*9.03	500x3
	CAMP	100	59877	7.90*7.90*9.26	500x3
	STIG	100	59329	7.82*7.82*9.32	500x3
	β- SITO	100	60056	7.91*7.91*9.18	500x3
HDC	CHOL	100	59816	7.83*7.83*9.39	500x3
	ERGO	100	59637	7.88*7.88*9.24	500x3
	CAMP	100	59882	7.78*7.78*9.52	500x3
	STIG	100	59319	7.86*7.86*9.25	500x3
	β-SITO	100	60037	7.88*7.88*9.29	500x3
DGT	CHOL	100	59836	7.84*7.84*9.34	500x3
	ERGO	100	59630	7.88*7.88*9.20	500x3
	CAMP	100	59899	7.83*7.83*9.36	500x3
	STIG	100	59330	7.86*7.86*9.18	500x3
	β-SITO	100	60057	7.87*7.87*9.26	500x3
OPD	CHOL	100	59813	7.89*7.89*9.22	500x3
	ERGO	100	59613	7.86*7.86*9.28	500x3
	CAMP	100	59885	7.87*7.87*9.26	500x3
	STIG	100	59316	7.91*7.91*9.09	500x3
	β-SITO	100	60043	7.77*7.77*9.52	500x3
**5 saponin-membrane (Saponins start from water)**					
5AHD	CHOL	100	59777	7.73*7.73*9.54	500x3
	ERGO	100	59580	7.93*7.93*9.11	500x3
	CAMP	100	59816	7.89*7.89*9.22	500x3
	STIG	100	59325	7.83*7.83*9.28	500x3
	β-SITO	100	60004	7.94*7.94*9.12	500x3
5AHN	CHOL	100	59738	7.94*7.94*9.05	500x3
	ERGO	100	59559	7.77*7.77*9.47	500x3
	CAMP	100	59789	7.82*7.82*9.36	500x3
	STIG	100	59265	7.91*7.91*9.09	500x3

	β-SITO	100	59959	7.88*7.88*9.23	500x3
5HDC	CHOL	100	59814	7.85*7.85*9.28	500x3
	ERGO	100	59614	7.90*7.90*9.14	500x3
	CAMP	100	59835	7.96*7.96*9.05	500x3
	STIG	100	59332	7.92*7.92*9.07	500x3
	β-SITO	100	59990	7.83*7.83*9.38	500x3
5DGT	CHOL	100	59803	7.85*7.85*9.28	500x3
	ERGO	100	59627	7.79*7.79*9.42	500x3
	CAMP	100	59806	7.84*7.84*9.29	500x3
	STIG	100	59336	7.88*7.88*9.10	500x3
	β-SITO	100	59979	7.89*7.89*9.22	500x3
5OPD	CHOL	100	59736	7.89*7.89*9.20	500x3
	ERGO	100	59515	7.92*7.92*9.09	500x3
	CAMP	100	59763	7.80*7.80*9.37	500x3
	STIG	100	59269	8.06*8.06*8.76	500x3
	β-SITO	100	59921	7.94*7.94*9.11	500x3
**5 saponin-membrane (Saponins start from membrane core region)**					
5AHD	CHOL	100	59777	8.01*8.01*8.91	500x3
	ERGO	100	59580	7.97*7.97*9.01	500x3
	CAMP	100	59816	7.95*7.95*9.05	500x3
	STIG	100	59325	7.98*7.98*8.94	500x3
	β-SITO	100	60004	7.83*7.83*9.36	500x3
5AHN	CHOL	100	59738	7.98*7.98*8.99	500x3
	ERGO	100	59559	8.08*8.08*8.78	500x3
	CAMP	100	59789	8.01*8.01*8.93	500x3
	STIG	100	59265	7.92*7.92*9.03	500x3
	β-SITO	100	59959	8.05*8.05*8.86	500x3
5DGT	CHOL	100	59803	7.88*7.88*9.21	500x3
	ERGO	100	59627	7.87*7.87*9.26	500x3
	CAMP	100	59806	7.93*7.93*9.13	500x3
	STIG	100	59336	7.93*7.93*9.01	500x3
	β-SITO	100	59979	7.97*7.97*9.01	500x3
5OPD	ERGO	100	59515	8.06*8.06*8.79	500x3

Table S3. Summary of stable binding events for neutral α-Hederin (AHD), deprotonated α-Hederin (AHN), and Hederacoside C (HDC) in single saponin–membrane systems. Values are reported as binding events/total simulations.

	AHD	AHN	DGT	HDC	OPD
DOPC/CHOL	1/3	2/3	3/3	1/3	2/3
DOPC/ERG	1/3	3/3	1/3	2/3	2/3
DOPC/CAMP	2/3	1/3	1/3	1/3	1/3
DOPC/SITG	3/3	0/3	0/3	0/3	2/3
DOPC/β-SITO	3/3	2/3	0/3	0/3	2/3
Total	10/15	8/15	515	4/15	9/15

Table S4. Summary of stable binding events for neutral α-hederin (AHD), deprotonated α-hederin (AHN), hederacoside C (HDC), digitonin (DGT), and ophiopogonin D (OPD) in five saponin–membrane systems. In these simulations, saponins were initially placed in the water above the bilayer. Values are reported as binding events/total amount of saponins.

	AHD	AHN	DGT	HDC	OPD
DOPC/CHOL	0/5	0/5	2/5	0/5	0/5
DOPC/ERG	0/5	0/5	2/5	0/5	0/5
DOPC/CAMP	0/5	0/5	1/5	0/5	0/5
DOPC/SITG	0/5	0/5	1/5	0/5	0/5
DOPC/β-SITO	0/5	0/5	0/5	0/5	0/5
Total	0/25	0/25	6/25	0/25	0/25

Movie S1. Interaction of α-hederin and hederacoside C with lipid membranes. Compared with the DOPC bilayer (left), the monodesmosidic protonated saponin α-hederin penetrates more deeply into the membrane in the presence of ergosterol (center). In contrast, the bidesmosidic saponin hederacoside C does not sufficiently penetrate the membrane core and instead remains on the surface of the lipid bilayer (right).

Movie S2. Interaction of saponins with DOPC/CHOL bilayers under multi-molecule conditions. Neutral α-hederin (left), deprotonated α-hederin (center), and hederacoside C (right) were initially pulled into the membrane by an external force during the first 5 s of the movie (corresponding to the first 20 ns of the simulation trajectory). The force was then removed, allowing spontaneous saponin–membrane interactions. Neutral α-hederin penetrates deepest into the membrane, whereas hederacoside C remains near the membrane surface and may dissociate into the water phase, consistent with the higher water affinity of bidesmosidic saponins.

S1. Raw data for in vivo yeast membrane lysis experiments.
Raw measurements from cell-based assays assessing yeast membrane lysis following treatment with saponins. Data correspond to the experiments (Nucleaocounter) presented in the main figures and include replicate measurements and experimental conditions used in the cell lysis assays.

S2. Raw data for in vitro large unilamellar vesicle (LUV) lysis assays.
Raw measurements from in vitro membrane permeabilization assays using large unilamellar vesicles (LUVs). Data correspond to the experiments (spectrophotmeter) shown in the main figures and include replicate measurements and conditions used to quantify vesicle lysis.

MD trajectories are available from the corresponding author upon reasonable request.

SI References

Sample References:

1. Bergman, L. W. Growth and Maintenance of Yeast BT - Two-hybrid systems — methods and protocols. Humana Press, 336 p., Two-hybrid systems — methods and protocols. Edited by Paul N. MacDonald, published by Humana Press, 2001, 336 p. 177, 9–14 (2001).
2. Sekula, B. C. & Nes, W. R. Metabolism of sterols by anaerobic Saccharomyces cerevisiae. Lipids 16, 195–198 (1981).
3. Achilles, J., Harms, H. & Müller, S. Analysis of living S. cerevisiae cell states - A three color approach. Cytometry Part A 69, 173–177 (2006).
4. Panaretou, B. & Piper, P. Isolation of yeast plasma membranes. Methods Mol Biol 313, 27–32 (2006).
5. Uzun, H.D., Vázquez-Hernández, M., Bandow, J. E. & Pomorski, T. G., In vitro Assay to Evaluate Cation Transport of Ionophores. Proteomics 22, 1–11 (2022).
6. Claereboudt, E. J. S., Eeckhaut, I., Lins, L. & Deleu, M. How different sterols contribute to saponin tolerant plasma membranes in sea cucumbers. Sci Rep 8, 1–11 (2018).
7. Quail, M. A. & Kelly, S. L. The Extraction and Analysis of Sterols from Yeast. in Yeast Protocols 123–132 (Humana Press, New Jersey). doi:10.1385/0-89603-319-8:123.
8. Khakimov, B., Motawia, M. S., Bak, S. & Engelsen, S. B. The use of trimethylsilyl cyanide derivatization for robust and broad-spectrum high-throughput gas chromatography-mass spectrometry based metabolomics. Anal Bioanal Chem 405, 9193–9205 (2013).
9. Trinh, M. D. L. et al. Site-directed genotype screening for elimination of antinutritional saponins in quinoa seeds identifies TSARL1 as a master controller of saponin biosynthesis selectively in seeds. Plant Biotechnol J 22, 2216–2234 (2024).
10. Jo, S., Kim, T., Iyer, V. G. & Im, W. CHARMM-GUI: A web-based graphical user interface for CHARMM. J Comput Chem 29, 1859–1865 (2008).
11. Jo, S., Lim, J. B., Klauda, J. B. & Im, W. CHARMM-GUI Membrane Builder for Mixed Bilayers and Its Application to Yeast Membranes. Biophys J 97, 50–58 (2009).
12. Wu, E. L. et al. CHARMM-GUI Membrane Builder toward realistic biological membrane simulations. J Comput Chem 35, 1997–2004 (2014).
13. Lee, J. et al. CHARMM-GUI Input Generator for NAMD, GROMACS, AMBER, OpenMM, and CHARMM/OpenMM Simulations Using the CHARMM36 Additive Force Field. J Chem Theory Comput 12, 405–413 (2016).
14. Hanwell, M. D. et al. Avogadro: an advanced semantic chemical editor, visualization, and analysis platform. J Cheminform 4, 17 (2012).
15. Kim, S. et al. CHARMM-GUI ligand reader and modeler for CHARMM force field generation of small molecules. J Comput Chem 38, 1879–1886 (2017).
16. Vanommeslaeghe, K. et al. CHARMM general force field: A force field for drug-like molecules compatible with the CHARMM all-atom additive biological force fields. J Comput Chem 31, 671–690 (2010).
17. Grubmüller, H., Heymann, B. & Tavan, P. Ligand binding: molecular mechanics calculation of the streptavidin-biotin rupture force. Science (1979) 271, 997–999 (1996).
18. Jarzynski, C. Nonequilibrium Equality for Free Energy Differences. Phys Rev Lett 78, 2690–2693 (1997).
19. Bonomi, M. et al. PLUMED: A portable plugin for free-energy calculations with molecular dynamics. Comput Phys Commun 180, 1961–1972 (2009).
20. Bonomi, M. et al. Promoting transparency and reproducibility in enhanced molecular simulations. Nat Methods 16, 670–673 (2019).
21. Tribello, G. A., Bonomi, M., Branduardi, D., Camilloni, C. & Bussi, G. PLUMED 2: New feathers for an old bird. Comput Phys Commun 185, 604–613 (2014).

22. Brooks, B. R. et al. CHARMM: The biomolecular simulation program. J Comput Chem 30, 1545–1614 (2009).
23. Huang, J. et al. CHARMM36m: an improved force field for folded and intrinsically disordered proteins. Nat Methods 14, 71–73 (2017).
24. Mark, P. & Nilsson, L. Structure and Dynamics of the TIP3P, SPC, and SPC/E Water Models at 298 K. J Phys Chem A 105, 9954–9960 (2001).
25. Evans, D. J. & Holian, B. L. The Nose–Hoover thermostat. J Chem Phys 83, 4069–4074 (1985).
26. Parrinello, M. & Rahman, A. Polymorphic transitions in single crystals: A new molecular dynamics method. J Appl Phys 52, 7182–7190 (1981).
27. Abraham, M. J. et al. GROMACS: High performance molecular simulations through multi-level parallelism from laptops to supercomputers. SoftwareX 1, 19–25 (2015).
28. Hess, B., Bekker, H., Berendsen, H. J. C. & Fraaije, J. G. E. M. LINCS: A linear constraint solver for molecular simulations. J Comput Chem 18, 1463–1472 (1997).
29. Darden, T., York, D. & Pedersen, L. Particle mesh Ewald: An N·log(N) method for Ewald sums in large systems. J Chem Phys 98, 10089–10092 (1993).
30. Steinbach, P. J. & Brooks, B. R. New spherical-cutoff methods for long-range forces in macromolecular simulation. J Comput Chem 15, 667–683 (1994).
31. Humphrey, W., Dalke, A. & Schulten, K. VMD: Visual molecular dynamics. J Mol Graph 14, 33–38 (1996).
32. Michaud-Agrawal, N., Denning, E. J., Woolf, T. B. & Beckstein, O. MDAnalysis: A toolkit for the analysis of molecular dynamics simulations. J Comput Chem 32, 2319–2327 (2011).
